# Transcriptomic profile of Pea3 family members reveal regulatory codes for axon outgrowth and neuronal connection specificity

**DOI:** 10.1038/s41598-020-75089-3

**Published:** 2020-10-23

**Authors:** Başak Kandemir, Gizem Gulfidan, Kazim Yalcin Arga, Bayram Yilmaz, Isil Aksan Kurnaz

**Affiliations:** 1 Institute of Biotechnology, Gebze Technical University, Kocaeli, Turkey; 2 Biotechnology Graduate Program, Yeditepe University, Kayisdagi, Istanbul Turkey; 3 Department of Bioengineering, Marmara University, Goztepe, Istanbul Turkey; 4 Faculty of Medicine, Yeditepe University, Kayisdagi, Istanbul Turkey; 5 Department of Molecular Biology and Genetics, Gebze Technical University, Kocaeli, Turkey; 6 Present Address: Department of Molecular Biology and Genetics, Baskent University, Ankara, Turkey

**Keywords:** Molecular biology, Transcriptomics, Neuronal development

## Abstract

PEA3 transcription factor subfamily is present in a variety of tissues with branching morphogenesis, and play a particularly significant role in neural circuit formation and specificity. Many target genes in axon guidance and cell–cell adhesion pathways have been identified for Pea3 transcription factor (but not for Erm or Er81); however it was not so far clear whether all Pea3 subfamily members regulate same target genes, or whether there are unique targets for each subfamily member that help explain the exclusivity and specificity of these proteins in neuronal circuit formation. In this study, using transcriptomics and qPCR analyses in SH-SY5Y neuroblastoma cells, hypothalamic and hippocampal cell line, we have identified cell type-specific and subfamily member-specific targets for PEA3 transcription factor subfamily. While Pea3 upregulates transcription of Sema3D and represses Sema5B, for example, Erm and Er81 upregulate Sema5A and Er81 regulates Unc5C and Sema4G while repressing EFNB3 in SH-SY5Y neuroblastoma cells. We furthermore present a molecular model of how unique sites within the ETS domain of each family member can help recognize specific target motifs. Such cell-context and member-specific combinatorial expression profiles help identify cell–cell and cell-extracellular matrix communication networks and how they establish specific connections.

## Introduction

**Figure 1:**
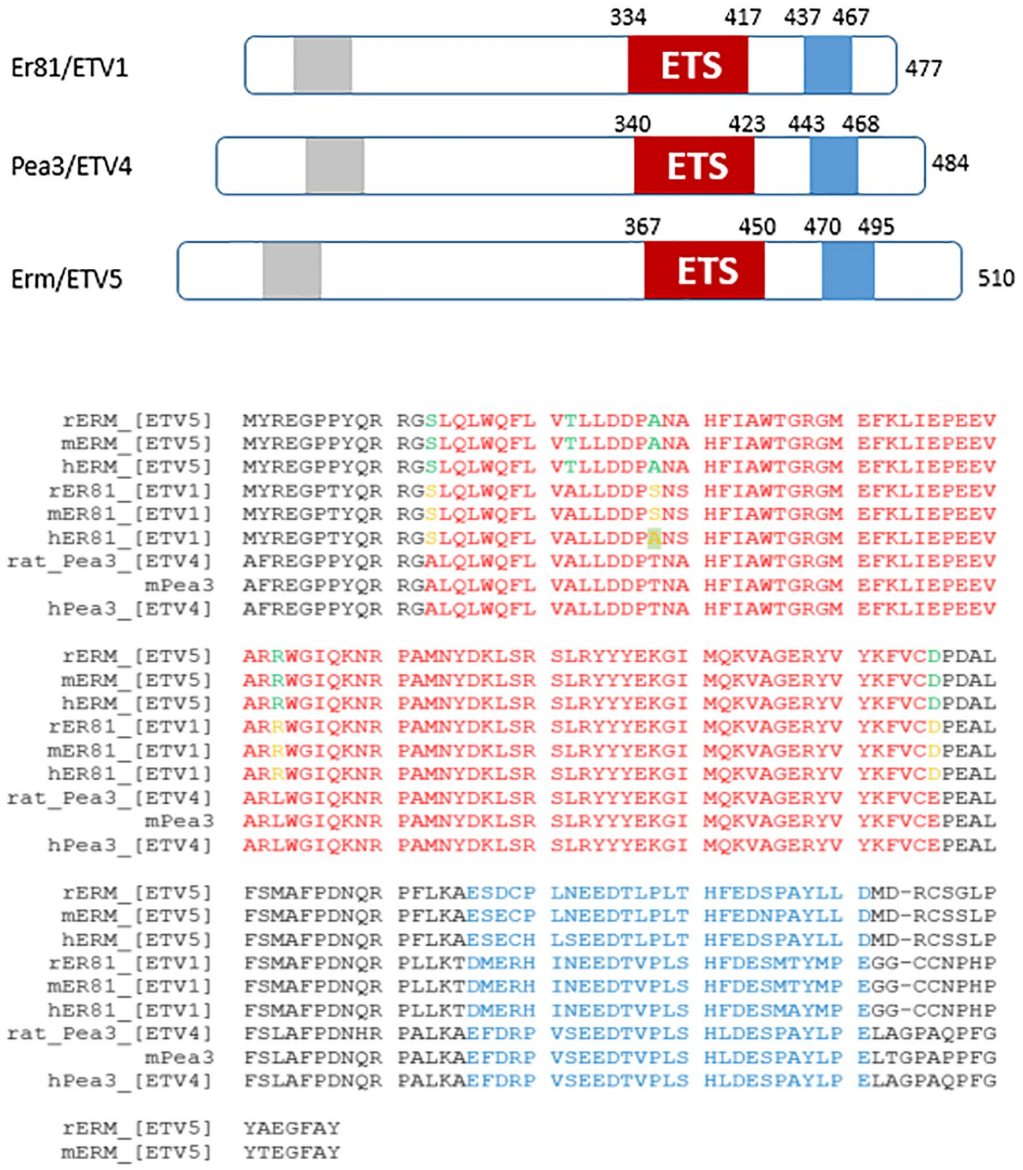
Schematic comparison of Pea3 family members’ domain structure and primary sequence of a region encompassing the ETS domain and the C terminal transactivation domain. Upper panel: Pea3 family members Er81/ETV1, Pea3/ETV4, and Erm/ETV5 have their DNA binding domain, the ETS domain, towards the C terminal (red box); all three members also contain two transactivation domains on each terminus (grey and blue boxes). Lower panel: Primary sequence of the region encompassing ETS domain (red font) and C terminal transactivation domain (blue font) across species (*r* rat, *m* mouse, *h* human); the ETS domain is well-conserved and the key residues that show variability among family members and species are indicated by green (those specific for ERM), yellow (those specific for Er81) font color.

ETS domain transcription factor superfamily is defined by a characteristic DNA binding domain, the ETS domain, which recognizes a core GGAA/T core motif in target promoters^1^, and is regulated by a number of inter- and intramolecular interactions^2^. Around 30 members of the ETS proteins have been identified in mammals, playing a role in different developmental processes. PEA3 family has been defined through a consensus ETS DNA binding domain towards its C terminus, and two transactivation domains at N and C termini. The ETS domain is highly conserved among Pea3 family members, with variations in only five positions within an 84 amino acid DNA binding domain (Fig. 1).

PEA3 proteins have been implicated in several different cancers, such as breast cancer^3–5^ and prostate cancer^6^. In normal development, PEA3 subfamily members are particularly found in tissues with branching morphogenesis such as kidney and lung^7^, motor neuron connectivity and dendritic arborization^8,9^, and retinal development^10^ as well as neuronal differentiation^11–13^. In hindbrain development, Fgf8 was found to induce Pea3 through the ERK MAPK pathway to specify isthmus formation^14,15^.

It has been shown that the late onset of PEA3 subfamily proteins is an essential requirement for normal sensory neuron differentiation^16^ and that the distal application of NGF in rat DRG sensory neurons was sufficient to induce expression of Pea3/Etv4 and Erm/Etv5^17^. Both Pea3/Etv4 and Erm/Etv5 were also reported to be essential for hippocampal dendritic arbor and spine formation through the BDNF signal^18^. In frontal cortex regionalization, Fgf17 and Emx2 have been shown to antagonistically regulate the expression of all three PEA3 family members^19^.

Since Pea3/ETV4 is extensively studied with respect to its metastatic function in many cancers, common target genes identified in these tissues were matrix metalloprotease enzymes, particularly MMP1, MMP2, and MMP9^3^. Other targets identified for Pea3/ETV4 has been vimentin^20^, the intercellular adhesion molecule ICAM-1^5,21^, osteopontin^22^, and vascular endothelial growth factor and cyclooxygenase-2^23^.

Very few genes have been studied concerning the role of PEA3 subfamily members in neurons: In *C. elegans,* ETS protein Ast-1 (axon steering defect-1) was shown to function in dopaminergic neuron differentiation through regulating dopaminergic genes^24^; cadherin-8, ephrin receptor 4 (Ephr4) and semaphorin-3E were shown to be Pea3 targets in neurons^9,13,25^. A large-scale analysis that used exogenously expressed constitutively active Pea3-VP16 fusion protein has also identified a large set of genes within cell adhesion, axon guidance, and nervous system development pathways such as ephrins and ephrin receptors, semaphorins, and cell adhesion molecules^13^.

PEA3 subfamily members Pea3, Erm, and Er81 are more than 95% identical within their DNA binding domain (ETS domain), with an overall 50% similarity^26^. Therefore, the main challenge has so far been not only identifying neuron-specific target genes, but rather the identification of family member-specific target promoters that justifies their role in forming specific neuronal connections.

In this study, we have addressed the problem through a global approach, using microarray in Pea3-, Erm- and Er81-transfected SH-SY5Y neuroblastoma and mHypo hypothalamic cell lines, as well as complementary qPCR analysis in the same cells as well as mHippo hippocampal cell lines. Analysis of transcriptome data has revealed several novel target genes unique for each family member in a cell type-specific manner, as well as genes that were regulated by more than one family member, or across cell types. We have further analyzed how such a well-conserved ETS domain can achieve such specific target gene regulation, and have identified certain residues within the ETS domain to be specific for either Pea3, Erm, or Er81, whereas certain residues are present in both Erm and Er81 or Er81 and Pea3. When known structures of DNA binding domains of each family member in complex with its cognate DNA was superimposed, and those specific residues were mapped, Ala326 and Leu3776 on Pea3 sequence was found to be in closer contact with DNA, whereas other unique regions that correspond to Ala345, Thr 351 and Glu419 on Pea3 structure faced away from DNA, suggesting those residues may be involved in member-specific protein interactions to achieve recognition of specific promoters. Differential regulation of certain cell surface proteins involved in axon guidance, migration, synapse refinement, dendritic morphology, and many other biological processes can explain how different Pea3 family members can achieve specificity in neuronal connectivity.

## Results

### Microarray-based target identification for each family member Pea3, Erm and Er81

It has been previously reported in the literature that subsets of motor neurons and muscle sensory afferents express Pea3 or Er81 almost exclusively and that functionally interconnected sensory and motor neurons express the same ETS variant^27^. It was intriguing that in this study motor and sensory neurons innervating the same target muscle expressed different Pea3 proteins, almost exclusively^27^. It was later shown that in *Pea3* mutant mice, motor neurons failed to innervate their target muscles, and the cell bodies of these neurons were mispositioned^9^, and that this function was through GDNF and Met signaling^28,29^. Recently, a transcriptomics study has revealed neuronal targets of the PEA3 family using a constitutively active Pea3-VP16^13^. Yet, it has not been clear how the specificity of functional motor neuron connectivity can be achieved through the expression of specific Pea3 family members that have such high homology within their DNA binding domain.

**Figure 2:**
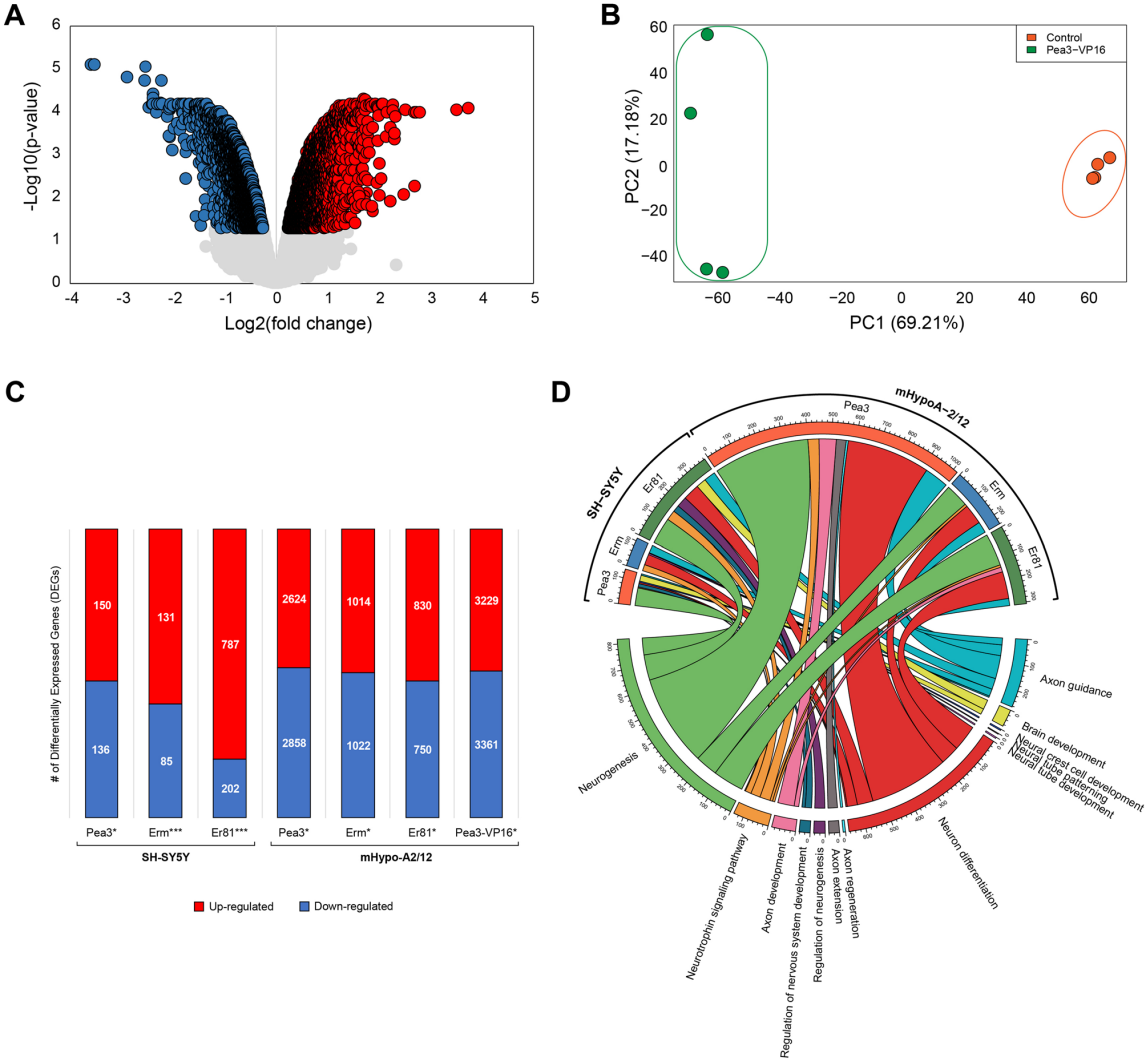
(**A**) Volcano plot showing the statistical significance (*p* value) versus fold change at logarithmic scale in transcriptome analysis for the mHypoA2/12 cells transfected with Pea3-VP16 expression vector with respect to the control cells. Up-regulated genes are shown in red, whereas down-regulated genes are shown in blue. *p* values < 0.05; LIMMA. (**B**) Principal Component Analysis graph for the mHypoA2/12 transfected with Pea3-VP16 expression vector. The variance explained by each principal component (PC) was represented as percentage in paranthesis. (**C**) Graphic depiction of the number of differentially expressed genes in SH-SY5Y and mHypoA2/12 cells transfected with Pea3, ERM or Er81 expression vectors, or mHypoA2/12 transfected with Pea3-VP16 expression vector. Up-regulated genes are shown in red, whereas down-regulated genes are shown in blue. *p* values: * < 0.05; *** < 0.001; LIMMA. (**D**) Circos plot for selected pathways related to neuronal development and function among enriched pathways in SH-SY5Y and mHypoA2/12 cells transfected with Pea3, ERM or Er81 expression vectors. The markers represent the number of genes enriched in each pathway. *p* values: < 0.05; Fisher’s Exact Test.

We have addressed this question through microarray analysis of two different model systems—SH-SY5Y neuroblastoma cells and mHypoA2/12 hypothalamic cell lines—that have been transfected to overexpress Pea3, Erm and Er81 proteins. The statistical analyses of transcriptome profiles yielded a set of differentially expressed genes (DEGs) and the high performance of the differential expression levels in discriminating the transfected cells from control cells was verified through Principal Component Analysis, as exemplified for the mHypoA2/12 transfected with Pea3-VP16 expression vector (Fig. 2A,B). We observed a modest number of DEGs under Pea3, Erm and Er81 overexpression (286, 216 and 989 genes, respectively), unlike the previous study where Pea3-VP16 was overexpressed in SH-SY5Y cells^13^, whereas significantly more genes have been responsive to Pea3, Erm and Er81 overexpression in hypothalamic mHypoA2/12 cells (5482, 2036 and 1580 genes, respectively; Fig. 2C). When mHypoA2/12 cells were transfected with Pea3-VP16, the number of DEGs was 6590 (Fig. 2C).

According to the gene set enrichment analyses as schematized in the circos plot, the nervous system-related pathways such as neuron development, neurogenesis, brain development, and axon guidance, among many others, were significantly enriched in Pea3-, Erm- and Er81-transfected SH-SY5Y (Fig. 2D, Table 1) as well as mHypoA2/12 cells (Fig. 2D, Table 2; see Supplemental Table S1 for full list). As can be seen in this plot, majority of the genes affected fall into neurogenesis and neuron differentiation pathways in both SH-SY5Y and mHypoA-2/12 cells transfected with Pea3, Erm and Er81; axon guidance pathway was also affected in both cell types, and additionally genes related to brain development, neural crest cell development and neural tube development were also regulated in SH-SY5Y cells transfected with Pea3 and Er81 (Fig. 2D). Although similar pathways were regulated to a similar extent in all cell types analyzed, some cell type-specific differences observed in target genes are expected to be due to different transcriptional partners in cells of different origin (SH-SY5Y is a neuroblastoma cell line used as a neuronal differentiation model, while mHypoA-2/12 is an immortalized hypothalamic cell line). Selected genes from these pathways have been chosen for further analysis, focusing on a subset of genes that are known regulators of axon growth and guidance, cell migration, synaptic connectivity, and dendritic morphology within Neurotrophin signaling & development and Axon Guidance pathways.

**Table 1:** Nervous system-related pathways that were chosen for further analysis among enriched pathways in Pea3-, Erm-, and Er81-transfected SH-SY5Y cells (including genes within the same pathways in mHypoA2/12 microarray).

	Term	Description	p value	# of Genes	Gene symbols
PEA3	GO:0051961	Negative regulation of nervous system development	2.2 × 10^–3^	14	BMPR1A,DLX2,ISL1,MED1,SYT4,ARHGEF2,ADGRG1,RAPGEF2,SEMA6C,PLK2,SEMA5B,ITM2C,HOOK3,SEMA3D
	GO:0048666	Neuron development	7.5 × 10^–3^	33	AGER,ADGRB1,BCL2,CTF1,ETV4,MNX1,ISL1,LHX1,LYN,MYH10,NEFM,NPTX1,PBX3,KLK6,SIAH2,SKIL,SYT4,LHX3, USP9X,RAPGEF2,SEMA6C,PLK2,IFT27,LZTS1,NEDD4L,SEMA5B,CLMN,ITM2C,SLITRK6,CCSAP,SEMA3D,FRYL,SKOR2
	GO:0048699	Generation of neurons	8.1 × 10^–2^	41	LZTS1, TUBB2B, SYT4, FRYL, USP9X, CTF1, ITM2C, HOOK3, SEMA5B, CDC42, CASP3, NPTX1, LHX1, BCL2, LHX3, SEMA3D, SKIL, NEDD4L, IFT27, RAPGEF2, NEFM, ETV4, KLK6, ARHGEF2, LYN, CLMN, HMG20B, ISL1, HMGA2, DLX2, SEMA6C, PLK2, NLGN4X, MNX1, RHEB, SIAH2, PBX3, SLITRK6, BMPR1A, MED1, MYH10
	GO:0014032	Neural crest cell development	8.3 × 10^–2^	5	SEMA5B, SEMA6C, SEMA3D, ISL1, BMPR1A
	GO:0022008	Neurogenesis	9.0 × 10^–2^	43	LZTS1, TUBB2B, SYT4, FRYL, USP9X, CTF1, ITM2C, HOOK3, CDC42, SEMA5B, CASP3, NPTX1, LHX1, BCL2, LHX3, SEMA3D, SKIL, NEDD4L, IFT27, RAPGEF2, NEFM, ETV4, KLK6, ARHGEF2, LYN, CLMN, HMG20B, ISL1, HMGA2, PRDM8, DLX2, SEMA6C, PLK2, MBOAT7, NLGN4X, MNX1, RHEB, SIAH2, PBX3, SLITRK6, BMPR1A, MED1, MYH10
	GO:0007420	Brain development	3.4 × 10^–2^	25	SYT4, HOOK3, CDC42, CASP3, LHX1, SHARPIN, BCL2, LHX3, SRD5A1, RAPGEF2, BCL2A1, FUT10, ISL1, HMGA2, TACC1, DLX2, NME5, MBOAT7, COX1, ARCN1, NLGN4X, H3F3B, MYH10, MED1, BMPR1A
	GO:0030182	Neuron differentiation		6	AIFM1, ATP7A, BCL6, DCLK1, DLX2, FRYL
	R-HSA-422475	Axon guidance		6	RHOC, MYH9, MYH10, CACNA1G, SHC2, MET
	path:hsa04722	Neurotrophin signaling		4	SHC2, NTRK3, PIK3R3, NFKBIB
ERM	path:hsa04722	Neurotrophin signaling		26	FOXO3, CRK, RELA, YWHAE, SHC2, RPS6KA3, BAD, NTRK3, PIK3R3, SHC1, RHOA, NFKBIB, BAX, PIK3R3, SHC1, RHOA, MAP3K5, RPS6KA3, IRS1, AKT1, BAD, NTRK2, MAP3K5, PIK3R3, SHC1, RHOA
	GO:0030182	Neuron differentiation		45	AGRN, AKT1, ALKBH1, ANAPC2, ARHGEF1, ATP7A, BLOC1S1, CAMK1, CAPZB, CDK5, CFL1, CSNK1D, DCLK1, DDR1, EFNB3, FLOT1, GDI1, HES1, ISL1, KDM1A, L1CAM, MAPK3, MAPK8IP2, MBD1, MICALL1, MYH10, NAPA, NLGN4X, NRCAM, PIN1, PLXNB2, PRKCSH, RAP1GAP, SLITRK6, SLC9A3R1, SPTAN1, SRGAP2, SSH2, SSH3, STAT3, STMN3, STXBP1, UNC13A, VEGFA, ZNF335
	R-HSA-422475	Axon guidance		32	MET, AP2A1, ARAF, RHOC, CFL1, COL6A2, CSNK2B, EFNB3, GRIN2D, L1CAM, MYH10, NRCAM, MAPK3, PSMC4, PSMD1, RAP1GAP, RPS6KA2, SHB, SHC1, SPTAN1, SPTBN2, VEGFA, CACNA1G, PSME3, PDLIM7, PSMF1, ARHGEF11, LYPLA2, PIP5K1C, SHC2, SPRED2, AGRN
	GO:0021915	Neural tube development	8.0 × 10^–2^	5	DVL2, SSBP3, FKBP8, PLXNB2, PRKACA
	GO:0021532	Neural tube patterning	6.1 × 10^–2^	3	SSBP3, FKBP8, PRKACA
ER81	GO:0051960	Regulation of nervous system development	6.0 × 10^–3^	28	ADGRB1,BCL2,BMPR1A,DLX2,ISL1,LHX1,LYN,MYB,MED1,KLK6,RHEB,SKIL,SYT4,TPBG,ARHGEF2,ADGRG1,RAPGEF2,CLSTN3,HMG20B,SEMA6C,PLK2,LZTS1,NEDD4L,SEMA5B,ITM2C,SLITRK6,HOOK3,SEMA3D
	path:hsa04722	Neurotrophin signaling		42	CRKL, PIK3CD, RELA, YWHAE, KIDINS220, RPS6KA3, MAPK14, CALM2, CALM3, NTRK3, PIK3R3, MAPK9, SHC1, RAC1, CAMK2B, SH2B3, MAGED1, AKT2, GRB2, PIK3R1, NFKBIB, CRK, MAPK13, NFKB1, MAPK12, NFKBIA, RPS6KA5, BRAF, PDPK1, PIK3CD, AKT1, CALM3, MAPK1, PIK3R3, PIK3R1, IRAK2, NFKB1, SHC1, CAMK2B, SH2B3, SOS1, GRB2
	GO:0048666	Neuron development	7.5 × 10^–3^	33	AGER,ADGRB1,BCL2,CTF1,ETV4,MNX1,ISL1,LHX1,LYN,MYH10,NEFM,NPTX1,PBX3,KLK6,SIAH2,SKIL,SYT4,LHX3, USP9X,RAPGEF2,SEMA6C,PLK2,IFT27,LZTS1,NEDD4L,SEMA5B,CLMN,ITM2C,SLITRK6,CCSAP,SEMA3D,FRYL,SKOR2
	R-HSA-422475	Axon guidance	1.2 × 10^–6^	48	AP2A1,ARAF,RHOC,CACNB3,CAMK2G,CDK5,CFL1,COL6A2,CSNK2B,EFNB3,GRIN2D,HRAS,HSP90AB1,L1CAM,MYH9,MYH10,NRCAM,PHB,PPP2R1A,MAPK3,MAPK7,PSMC2,PSMC4,PSMD1,PSMD3,RAP1GAP,RPS6KA2,SHB,SHC1,SPTAN1,SPTBN2,TLN1,VEGFA,CACNA1G,PDLIM7,PSMF1,ARHGEF11,RASA4,PSME3,CAP1,LYPLA2,PIP5K1C,SHC2,DOK4,ARHGAP39,TUBA1C,SPRED2,AGRN
	GO:0007420	Brain development	3.7 × 10^–2^	42	NAPA, MDK, CITED1, HOOK3, MEN1, SEZ6L2, KDM1A, BAK1, ZNF148, MAPKAP1, SEC16A, PTN, H2AFX, HSPA5, CDK5RAP2, ATP6V0D1, PLCB1, SDF4, SMG9, SSBP3, PLXNB2, CST3, ROGDI, ARID1A, ISL1, CDK5, SIRT2, ZNF335, ATP7A, HES1, NCOA1, G6PD, TRAPPC9, MBOAT7, H2AFY2, NLGN4X, SPTBN2, YWHAQ, PYGO2, NRGN, SRGAP2, MYH10
	GO:0050767	Regulation of neurogenesis	1.6 × 10^–2^	44	HMGB2, RAP1GAP, SSH3, SSH2, L1CAM, PRKCSH, TGFB1, HOOK3, NRCAM, AKT1, KDM1A, PRMT5, OBSL1, PTN, NCKIPSD, CDK5RAP2, SCRT1, CHRNA3, ANAPC2, GDI1, ARHGEF2, ARHGEF1, PLXNB2, RELA, LGALS1, SF3A2, ISL1, CDK5, MBD1, SIRT2, STAT3, ZNF335, HES1, EIF4G2, NCOA1, CSNK1D, CFL1, VEGFA, CPNE1, CAMK1, WDR1, APBB1, UNC13A, SRGAP2
	GO:0048699	Generation of neurons	1.9 × 10^–2^	78	HRAS, L1CAM, PRKCSH, TGFB1, HOOK3, NRCAM, AKT1, KDM1A, PRMT5, CDK5RAP2, NCKIPSD, IFT27, SCRT1, CHRNA3, ANAPC2, ARHGEF2, ARHGEF1, EFNB3, STMN3, PLXNB2, RELA, FLOT1, STXBP1, SLC9A3R1, MBD1, CDK5, ZNF335, HOXD9, HES1, DDR1, EIF4G2, NCOA1, TRAPPC9, CFL1, VEGFA, MAPK3, C1QL1, USP21, CAMK1, SLITRK6, KIF26A, UNC13A, SRGAP2, HMGB2, RAP1GAP, FRYL, SSH3, SSH2, NAPA, CAPZB, PIN1, LAMB2, BLOC1S1, ETV1, PTN, OBSL1, AGRN, LRFN4, GDI1, LGALS1, ISL1, SF3A2, STAT3, SIRT2, MICALL1, ATP7A, GBA2, CSNK1D, NLGN4X, MAPK8IP2, CPNE1, SPTBN2, ALKBH1, WDR1, MAP6, APBB1, MYH10, SPTAN1
	GO:0030182	Neuron differentiation	4.1 × 10^–2^	69	HRAS, L1CAM, PRKCSH, NRCAM, AKT1, KDM1A, CDK5RAP2, NCKIPSD, IFT27, CHRNA3, ANAPC2, ARHGEF1, EFNB3, STMN3, PLXNB2, FLOT1, STXBP1, SLC9A3R1, MBD1, CDK5, ZNF335, HOXD9, HES1, DDR1, EIF4G2, NCOA1, TRAPPC9, CFL1, MAPK3, VEGFA, C1QL1, USP21, CAMK1, KIF26A, SLITRK6, UNC13A, SRGAP2, RAP1GAP, FRYL, SSH3, SSH2, NAPA, CAPZB, PIN1, LAMB2, BLOC1S1, ETV1, PTN, OBSL1, AGRN, LRFN4, GDI1, LGALS1, ISL1, SF3A2, STAT3, MICALL1, ATP7A, GBA2, CSNK1D, NLGN4X, MAPK8IP2, CPNE1, SPTBN2, ALKBH1, MAP6, APBB1, SPTAN1, MYH10

*GO* gene ontology, *R-HSA* reactome, *path:hsa* KEGG term.

**Table 2:** Selected pathways related to neuronal development and function among enriched pathways in Pea3-, Erm- and Er81-transfected mHypoA-2/12 cells.

	Term	Description	p-Value	# of genes
Pea3	GO:0022008	Neurogenesis	2.43 × 10^–3^	401
	GO:0030182	Neuron differentiation	7.21 × 10^–3^	348
	path:hsa04722	Neurotrophin signaling pathway	5.95 × 10^–3^	46
	GO:0048675	Axon extension	2.17 × 10^–2^	41
	GO:0061564	Axon development	1.36 × 10^–3^	72
	path:hsa04360	Axon guidance	1.30 × 10^–3^	35
	R-HSA-422475	Axon guidance	3.72 × 10^–3^	77
	GO:0007411	Axon guidance	2.00 × 10^–2^	35
	GO:0031103	Axon regeneration	4.19 × 10^–2^	9
Erm	GO:0022008	Neurogenesis	4.40 × 10^–5^	98
	GO:0030182	Neuron differentiation	1.69 × 10^–4^	84
	R-HSA-422475	Axon guidance	9.80 × 10^–6^	47
	path:hsa04722	Neurotrophin signaling pathway	1.36 × 10^–2^	11
Er81	GO:0022008	Neurogenesis	2.67 × 10^–7^	134
	GO:0030182	Neuron differentiation	1.20 × 10^–5^	113
	R-HSA-422475	Axon guidance	1.95 × 10^–3^	26
	path:hsa04360	Axon guidance	1.39 × 10^–2^	11
	GO:0061564	Axon development	3.82 × 10^–2^	20
	path:hsa04722	Neurotrophin signaling pathway	1.15 × 10^–2^	10

*GO* Gene ontology, *R-HAS* Reactome, *path:has* KEGG term.

### qPCR validation of selected targets in SH-SY5Y, mHypoA2/12, and mHippoE-14 cell lines

Functionally interconnected neurons expressing the same transcription factor can only recognize each other through their cell surface proteins or secreted molecules. We have therefore concentrated on growth factors, growth factor receptors, chemoattractant, and chemorepellent molecules, as well as regulators thereof, in our qPCR validation assays. To that end, we have transfected mHypoA2/12 cells with expression vectors for Pea3, Erm, or Er81, and analyzed the expression of genes identified in microarray studies.

**Figure 3:**
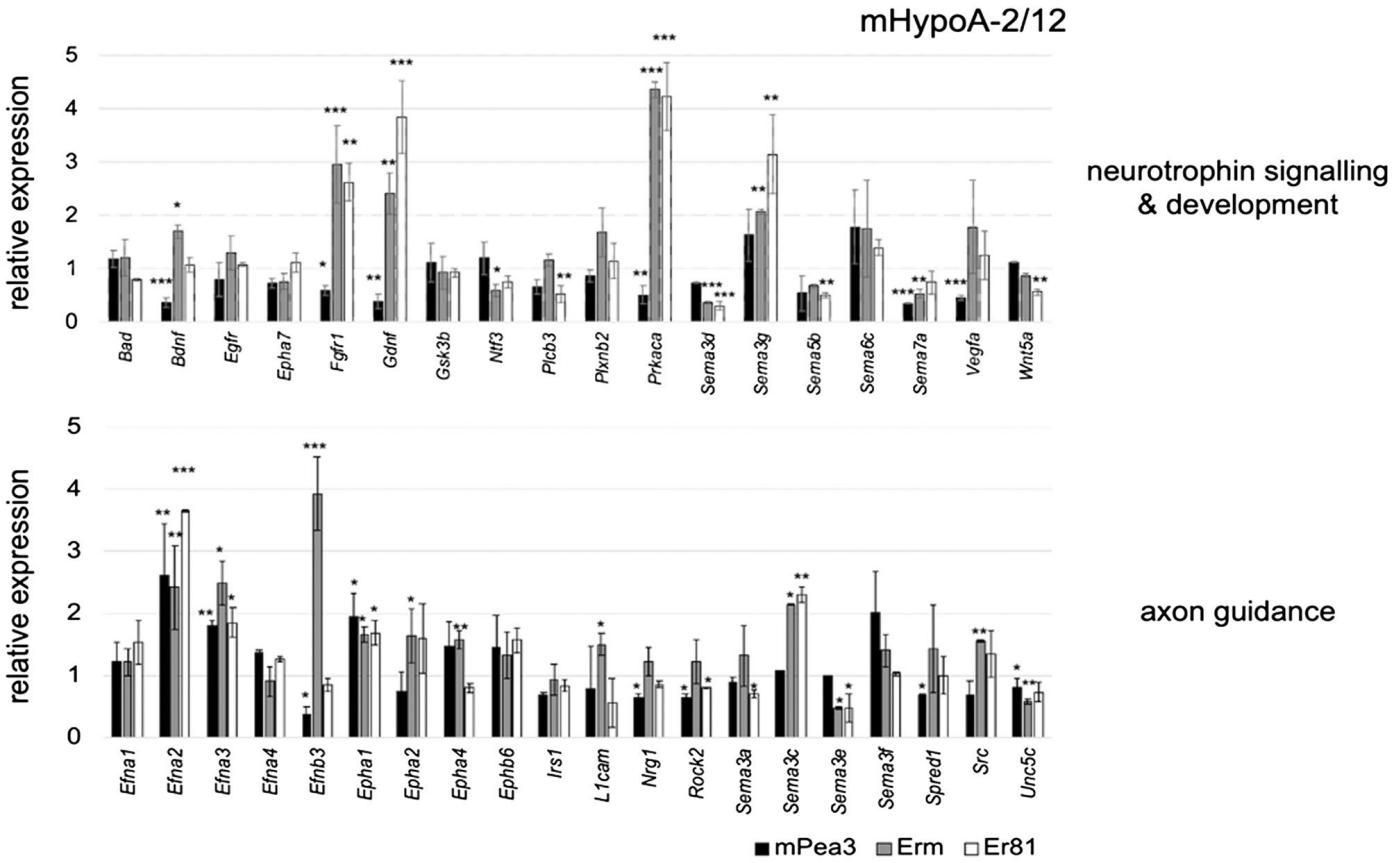
qPCR validation analysis of selected genes in Neurotrophin signaling & development pathway (upper panel) and Axon Guidance pathway (lower panel) in mHypoA2/12 cells transfected with Pea3, Erm or Er81 expression vectors. The reaction was standardized using *Gapdh* and *β-Actin* as housekeeping genes and mRNA expression level of each gene was shown as relative expression as compared to control (pCDNA3 transfected cells) group. Values are expressed as mean ± SEM. *p* values: * < 0.05; ** < 0.01; *** < 0.001; Student’s t-test.

Among the Neurotrophin signaling & development pathway, *Fgfr1* was downregulated by Pea3, but upregulated by Erm and Er81, while *Egfr* did not show a significant change (Fig. 3, upper panel). *Vegfa* was repressed by Pea3, but not significantly changed in Erm or Er81 overexpression, whereas *Gdnf* was repressed by Pea3 and activated over twofold by Erm and over threefold by Er81 (Fig. 3, upper panel). *Bdnf*, on the other hand, was repressed by Pea3 and activated by Erm, but not significantly changed by Er81, while *Ntf3* was repressed by Erm and not affected by others and *Wnt5a* expression was repressed by Er81 (Fig. 3, upper panel). Among genes related to Neurotrophin signaling, proliferation and apoptosis-associated genes were also found: regulation of pro-apoptotic gene *Bad* or glycogen synthase kinase 3 beta, *Gsk3b*, by either family members was not validated, however phospholipase C beta (*plcb3*) downstream of G protein-coupled receptor signaling was repressed by Er81, while *Prkaca*, the catalytic subunit of protein kinase A, was repressed by Pea3 and upregulated almost fourfold by both Erm and Er81 (Fig. 3, upper panel).

Semaphorins, some of which had previously been identified as a target for Pea3-VP16, also showed differential regulation by family members: *Sema5b* was repressed by Er81 but not others, while *Sema3d* was repressed by both Erm and Er81; *Sema7a* was repressed by both Pea3 and Erm, while *Sema3g* was upregulated by both Erm and Er81 (Fig. 3, upper panel). *Sema6c* and *EphA7* were not validated as a genuine target for Pea3 family members in mHypoA2/12 cells.

Among genes selected within the Axon Guidance pathway, *Spred1*, a member of the Sprouty family of proteins that interact with tyrosine kinases and inhibit growth factor-mediated activation of MAPKs, was repressed by Pea3 but not altered by Erm or Er81 (Fig. 3, lower panel). *Src* kinase known to regulate cytoskeletal organization, critical for migration and axon outgrowth, is upregulated by Erm and Er81 but not Pea3, whereas *Rock2* kinase, which is involved in actin cytoskeleton organization and neurite retraction, was repressed by both Pea3 and Er81 but not Erm (Fig. 3, lower panel). Cell adhesion molecule *L1cam* was activated by Erm but not others, and neuregulin 1, *Nrg1*, that is a ligand for EGF Receptors ERBB3 and ERBB4, was repressed by Pea3 but not others (Fig. 3, lower panel).

There was also differential regulation among ephrins, ephrin receptors, and semaphorins: *Epha2* and *Epha4* were upregulated by Erm but not others, while *Efnb3* was repressed by Pea3 but upregulated nearly fourfold by Erm (Fig. 3, lower panel). *Sema3a* was repressed by Er81, and *Sema3e* was repressed by both Erm and Er81, while *Sema3c* was upregulated by Erm and Er81; *Unc5c* was repressed by both Pea3 and Erm (Fig. 3, lower panel). Some were regulated by all three proteins: *Epha1, Efna2,* and *Efna3* were upregulated by all Pea3, Erm and Er81. *Irs1, Efna1,* and *Efna4* were not validated as genuine targets of this ETS subfamily in mHypoA2/12 cells.

**Figure 4:**
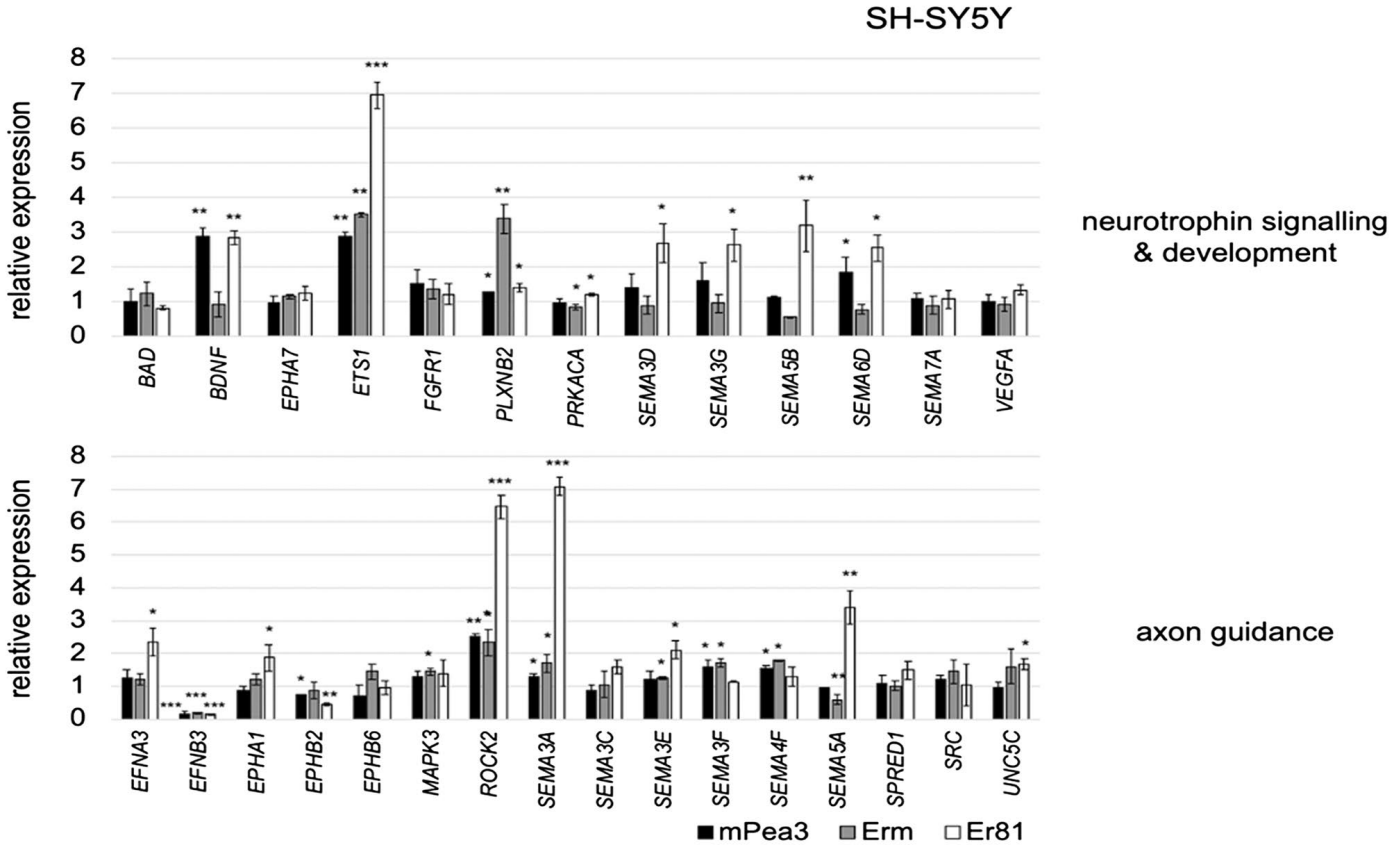
qPCR validation analysis of selected genes in Neurotrophin signaling & development pathway (upper panel) and Axon Guidance pathway (lower panel) in SH-SY5Y cells transfected with Pea3, Erm or Er81 expression vectors. The reaction was standardized using *Gapdh* and *β-Actin* as housekeeping genes and mRNA expression level of each gene was shown as relative expression as compared to control (pCDNA3 transfected cells) group. Values are expressed as mean ± SEM. *p* values: * < 0.05; ** < 0.01; *** < 0.001; Student’s t-test.

Similar validation was repeated for SH-SY5Y cells overexpressing Pea3, Erm, and Er81 proteins, covering a similar subset of genes. *BAD, FGFR1, VEGFA, EPHA7,* and *SEMA7A* among the Neurotrophin signaling & development pathway could not be validated by qPCR. *BDNF* and *PRKACA* were regulated by Pea3 and Er81, and another ETS gene, *ETS1* was upregulated by all three Pea3 family members (Fig. 4, upper panel). Semaphorin receptor, *PLXNB2*, was upregulated by Pea3 and Erm, *SEMA6D* was upregulated by Pea3 and Er81, and *SEMA3D, SEMA3G,* and *SEMA5B* were upregulated by Er81 only (Fig. 4, upper panel).

Regarding Axon Guidance pathways in SH-SY5Y microarray data, *SRC, SPRED1, SEMA3C,* and *EPHB6* were not validated by qPCR. *EFNA3, EPHA1*and *UNC5C* were upregulated only by Er81; *EFNB3* was repressed, while *ROCK2* and *SEMA3A* were activated by all three Pea3 proteins; *EPHB2* was repressed by Pea3 and Er81, *MAPK3* was upregulated by Erm, *SEMA3E* was upregulated by Erm and Er81, whereas *SEMA5A* was repressed by Erm and activated by Er81 (Fig. 4; lower panel). *SEMA3F* and *SEMA5F* were activated by Pea3 and Erm (Fig. 4, lower panel).

**Figure 5:**
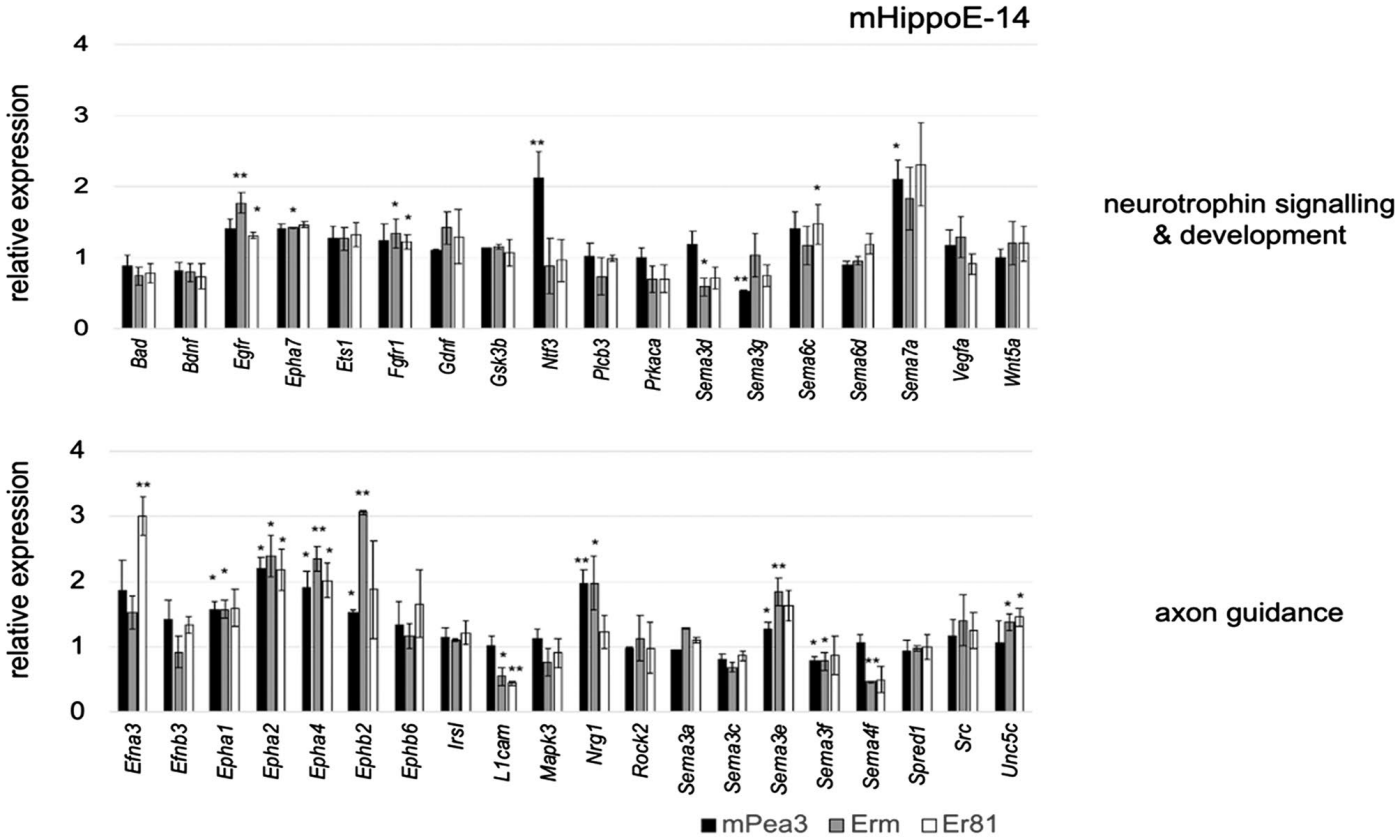
qPCR analysis of selected genes in Neurotrophin signaling & development pathway (uppr panel) and Axon Guidance pathway (lower panel) in mHippoE-14 cells transfected with Pea3, Erm or Er81 expression vectors. The reaction was standardized using *Gapdh* and *β-Actin* as housekeeping genes and mRNA expression level of each gene was shown as relative expression as compared to control (pCDNA3 transfected cells) group. Values are expressed as mean ± SEM. *p* values: * < 0.05; ** < 0.01; *** < 0.001; Student’s t-test.

Finally, we have checked a third cell type, mHippoE-14 hippocampal cell line, for the regulation of these putative targets identified by microarray analysis. Among the significantly regulated genes in Neurotrophin signaling & development pathway, *Fgfr1* and *Egfr1* were upregulated by both Erm and Er81, while *Ntf3, Sema3g,* and *Sema7a* were regulated by Pea3 only, *Sema6c* was regulated by Er81 only, and *Sema3d* and *Epha7* were regulated by Erm only (Fig. 5, upper panel). Among the significantly regulated genes in Axon guidance pathway, *Epha2* and *Epha4* were regulated by all three family members; *Epha1, Ephb2, Sema3e, Sema3f.,* and *Nrg1* were regulated by both Pea3 and Erm; *L1cam* and *Unc5c* were regulated by both Erm and Er81; *Sema4f.* was regulated by Erm only, and *Efna3* was regulated by Er81 only (Fig. 5, lower panel).

Differential regulation of these selected genes by different Pea3 family members in SH-SY5Y cells and mHypoA2/12 cells in microarray vs qPCR analyses have been summarized in Supplemental Tables S2 and S3.

### Confirmation of Pea3 binding to a selected subset of identified promoters

Having established a family member-specific and cell context-dependent target identification of Pea3 subfamily, we have next addressed whether those promoters contained high-affinity binding motifs. To that end, we have first analyzed a series of promoters of putative targets identified in microarray analyses. Among the promoters sequences that could be retrieved from databases, a selected subset was scanned with Promo3.0 for Pea3 binding motif and dissimilarity to consensus site, given as % dissimilarity score (dissimilarity score of less than 5% is accepted as a potential binding site) or JASPAR (Supplemental Table S4), as described in “Materials and methods”.

**Figure 6:**
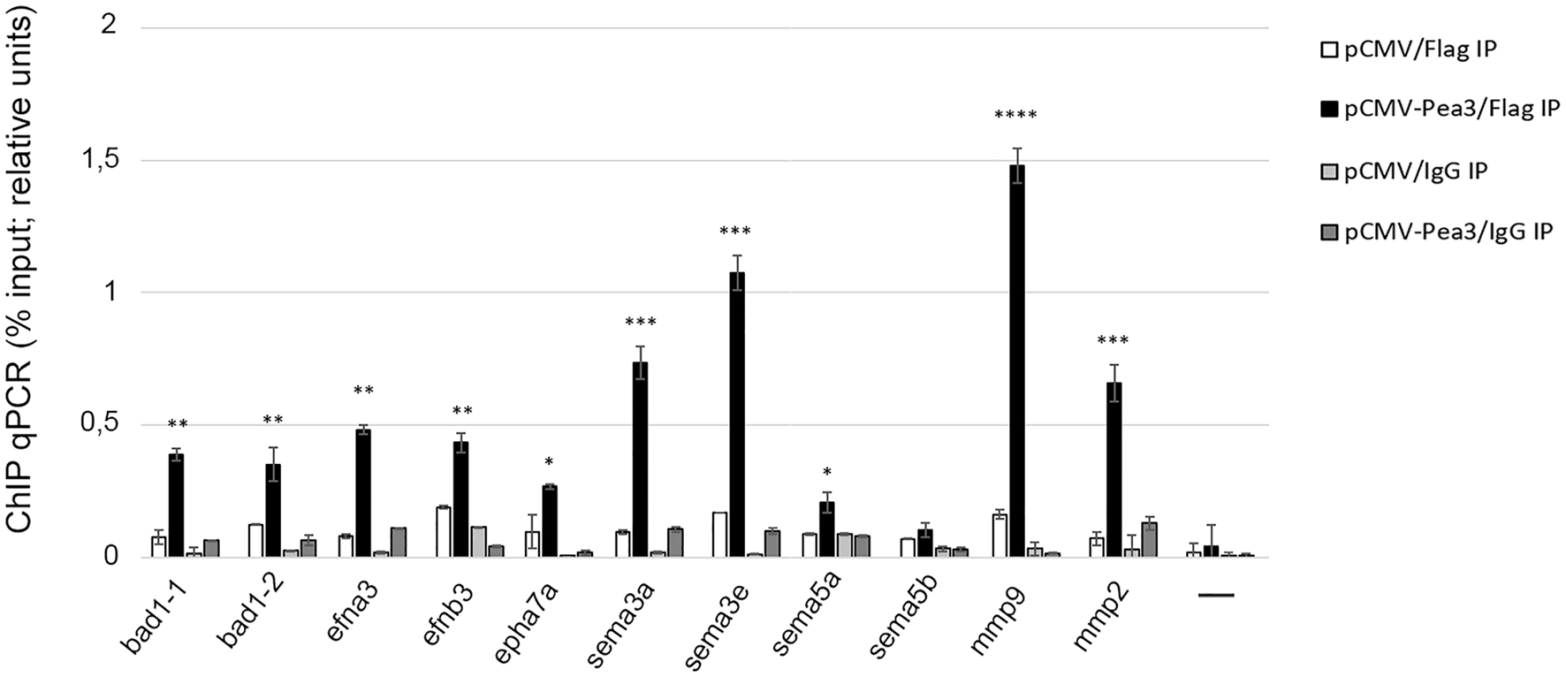
Chromatin IP (ChIP) analysis of selected promoters for Pea3 binding. SH-SY5Y cells were transfected either with pCMV or pCMV-Pea3 and cell lysates were immunoprecipitated with either Flag antibody (Flag IP) or IgG (IgG IP), followed by ChIP-qPCR for the indicated promoter motifs. The error bars show the SEM for 2 independent experiments. *p* values: * < 0.05; ** < 0.01; *** < 0.001; **** < 0.0001; ANOVA (one way).

Based on these dissimilarity scores, we have next carried out chromatin immunoprecipitation (ChIP) assay to determine whether Pea3 protein does indeed bind to its potential target promoters. To that end, we have transfected SH-SY5Y cells with Flag-tagged Pea3 expression vector, and immunoprecipitated using Flag antibody. When precipitated DNA was analyzed with qPCR, Pea3 was found to bind *efna3* promoter, which was found to be upregulated by all three Pea3 family members, but not *sema5b*, which was found to be regulated by Er81 in mHypoA2/12 and SH-SY5Y cells (see also Figs. 3, 4, and 6). *Sema5a*, which was previously shown to be regulated by Erm and Er81 but not Pea3 in SH-SY5Y cells (see Fig. 4) showed a modest albeit significant binding to Pea3; similarly, *bad* promoter, whose regulation could not be validated by qPCR in any of the three cell types, and *Epha7*, which responded to Erm overexpression in mHippoE-14 cells, showed modest binding by Pea3-Flag relative to control (Fig. 6). *Efnb3*, *sema3a,* and *sema3e*, which have been shown to respond to Pea3 overexpression in a cell context-dependent manner, were found to bind to Pea3 in ChIP assay (Fig. 6). *Mmp9* and *mmp2* were used as a positive control; no DNA reaction was used as a negative control.

## Discussion

The role of Pea3 family members in neuronal connectivity has been largely studied in spinal motor neuron subsets, where researchers have shown that an intrinsic program of ETS expression pattern, in particular, that of Pea3 and Er81, coordinates motor neuron cell body positioning as well as terminal arborization^9^. It was furthermore shown that functionally interconnected motor and sensory neurons that innervate the same muscle expressed—almost exclusively—either Pea3 or Er81^27^, indicating that the specific family members somehow instruct the neuron where to migrate to and which target to connect with. It should be noted, however, that this does not mean Pea3 family members are the only transcription factors involved in target selectivity and neuronal circuit formation, as axonal elongation, target identification and circuit formation are complex and multi-step processes, involving various signal transduction and axonal guidance pathways, gap junctions and other cell–cell communication molecules. It has been shown, however, that for example Er81- sensory neurons do not innervate Er81 + motor neurons and there appears to be exclusive expression of Pea3 family members in different motor neuron subgroups^27^; furthermore, the function of Pea3 family members is not limited to the spinal motor neurons and sensory neurons: Pea3 family members Pea3 and Erm have been shown to play a role in dendritic arborization in a Bdnf-dependent manner^18^; Er81 and CaMKIV together have been shown to be important for dopaminergic determination during the migration of progenitors arising from the olfactory bulb; Er81 was found to be expressed in ventricular zone at E15 and in prospective layer V neurons projecting to the spinal cord^30^, however, whether it is specifically required to distinguish targets and achieve specific connectivity is yet to be studied.

A transcriptomic study using constitutively expressed Pea3-VP16 had identified a large set of neuronal-related pathways and genes regulated by Pea3^13^, but a thorough analysis of the unique targets of each Pea3 family member has been elucidated for the first time in this study. We have identified targets in five categories: (a) those regulated by all three family members, (b) those regulated by Pea3 and Er81, (c) those regulated by Pea3 and Erm, (d) those regulated by Erm and Er81, and (e) those unique for each family member (supplemental Tables S1 and S5). These were found to be largely overlapping between SH-SY5Y and mHypoA2/12 cells, although a larger set of genes within these pathways were differentially regulated in mHypoA2/12 cells (Supplemental Table S1).

**Figure 7:**
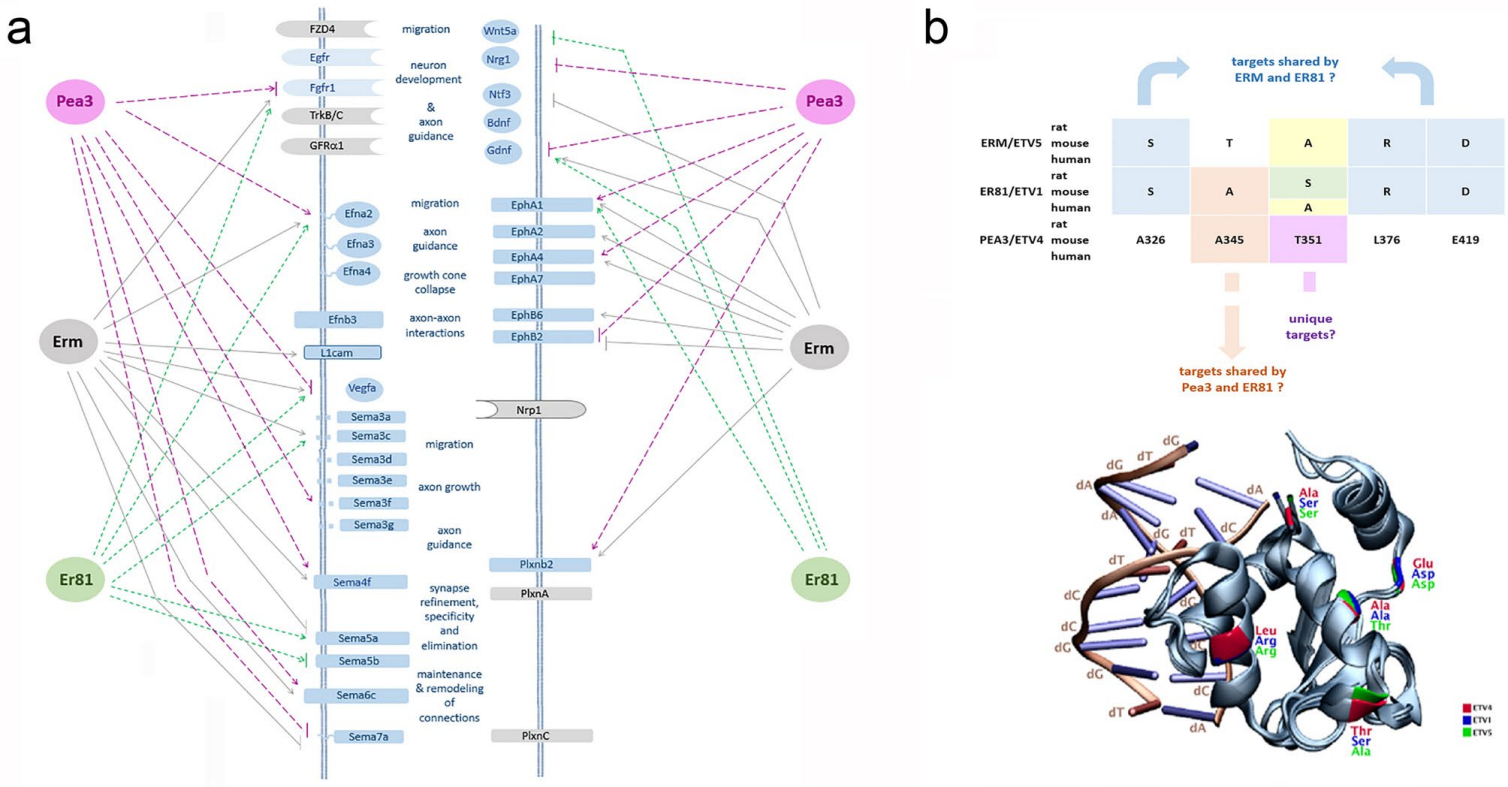
(**a**) Schematic model of selective regulation of a subset of cell surface proteins involved in migration, neuron development, axon guidance and other pathways by Pea3 family members. Not every regulation is depicted for the sake of simplicity; where a family member can activate or repress the indicated target depending on the cellular context, both regulations have been shown simultaneously. Pea3 regulations were shown as dashed pink lines and arrows, Erm regulations were shown as grey lines or arrows, and Er81 regulations were shown as green dotted lines or arrows. Those cell surface molecules that were experimentally shown to be regulated by Pea3 family members appear as blue shapes, whereas known interaction partners that have not been shown to be regulated by Pea3 family members appear as grey shapes. (**b**) Schematic summary of differences within the ETS domain among Pea3 family members (upper panel); and superimposition of molecular models for three Pea3 family members bound to cognate DNA (lower panel; see text for details); structures were visualized via Visual Molecular Dynamics (VMD), molecular visualization program, (Version 1.9.3; URL: https://www.ks.uiuc.edu/Research/vmd/)^43^. The structures of Etv1, Etv4 and Etv5 were obtained from the PDB database with PDB IDs of 4BNC, 4UUV, and 4UNO, respectively.

When genes coding for cell surface or secreted proteins were specifically considered, it was evident that particular combinations of ligands and receptors or cell–cell and cell-extracellular matrix interacting proteins were differentially regulated by individual Pea3 family members or pairs of Pea3 proteins. Wnt5a, which binds frizzled receptor FZD4 and promotes dendrite development and dendritic morphogenesis^31^, is repressed by Pea3 and Er81, but not Erm (Fig. 7a). Sema5b, which is shown to be important for synapse elimination in hippocampal neurons^32^, is regulated by Er81 in both SH-SY5Y and mHypoA2/12 in opposite directions (Fig. 7a). Sema3f., on the other hand, which is critical for limbic system circuitry development^33^, was regulated by Pea3 and Erm in mHippoE-14 and SH-SY5Y cells (Fig. 7a). PlexinB2 (Plxnb2) that has been reported to be a receptor for semaphorins 4a, 4c, 4d and 4g in the context of neuronal axon guidance and cell migration events, is regulated by both Pea3 and Erm (Fig. 7a). We propose that by differentially regulating either the ligands, receptors, or both in corresponding cell types, Pea3 family members ensure that the cells expressing them recognize other cells that express the same Pea3 family member through a “barcode” of cell surface and secreted proteins. We believe this study is a first step towards deciphering this transcriptional code, although, in order to get a more complete picture to analyze motor neuron and other circuitries, more detailed single-cell RNAseq analyses with each neuron in the circuit of interest should be conducted in the future.

Such differential targeting of promoters by each family member can help explain the selectivity of the circuit components; however, it was still unclear how proteins with such highly similar DNA binding domains could distinguish between promoters to ensure this transcriptional barcode. To that end, we have reverted to the five categories of target genes (Supplemental Tables S4 and S6) and addressed whether the small variations within the ETS domain (Fig. 1) corresponded to these categories. When the 5 residues that make up the ETS domain variations among family members were aligned (numbering given only for Pea3/ETV4), a certain profile became evident: Residue corresponding to A326 in Pea3 was changed to Serine in Erm and Er81; similarly residues corresponding to L376 and E419 in Pea3 were Arginine and Aspartate, respectively, in both Erm and Er81, indicating those residues may account for target recognition by both Erm and Er81 vs unique targets of Pea3 (Fig. 7b, upper panel). On the other hand, residue corresponding to A345 in Pea3 was again Alanine in Er81, but changed to a Threonine in Erm, indicating this particular residue may be important for common target recognition by Pea3 and Er81, and unique sites for Erm (Fig. 7b, upper panel). Finally, residue corresponding to T351 in Pea3 sequence was converted to Alanine in Erm, but was changed to Alanine in human Er81 and Serine in mouse and rat Er81, indicating not only that this may account for unique sites for Erm, Er81, and Pea3 in most mouse, rat, and human, but also for differential gene regulation by Er81 in human vs rodent systems, which is not observed in any other family member (Fig. 7b, upper panel).

To correlate these findings to DNA binding of their ETS domain structures, we have then carried out molecular modeling, where structures of Etv1 and Etv5 without DNA were aligned to the structure of Etv4 with DNA using PyMol software, as described in Materials and Methods. We have then mapped the residues identified in the ETS domain onto this superimposed structure (Fig. 7b, lower panel). The residues corresponding to A326 and L376 in Pea3 were found to be in close proximity to the cognate DNA, indicating that these two residues may specifically interact with Pea3 binding sites containing GGAA/T core motif on target promoters, and account for the differential target recognition by Erm and Er81 vs Pea3 (Fig. 7b, lower panel). The remaining residues corresponding to A345, T351, and E419 on Pea3, however, all resided away from the DNA, indicating that differential target recognition by Er81 and Pea3, or unique target recognition by each family member, may not be through selective binding to promoters, but rather through selective protein–protein interaction that may indirectly affect recruitment and/or activation of those specific targets (Fig. 7b, lower panel).

In this study, we have presented a global transcriptomic study to identify novel targets of Pea3 family members so as to explain how such highly homologous transcription factors could affect selective circuit formation during development. We have also shown that target recognition is dependent on the cellular context, using SH-SY5Y, hypothalamic mHypoA2/12 and hippocampal mHippoE-14 cells, which showed that there is likely a transcriptional barcode that affects the distribution of cell surface proteins, and ligand-receptor pairs on different neurons could help identify the correct and specific target for each neuron. We have furthermore presented a model whereby the small variations within the DNA binding domain may account for the differential regulation of the target promoters identified. It will also be interesting in the future to study whether a similar transcriptional barcode is present for target identification in other tissue types where Pea3 family proteins are involved in branching morphogenesis, such as kidney and lung.

## Materials and methods

### Cell culture and transfection

SH-SY5Y human neuroblastoma cell line (ATCC CRL-2266) and mHypoA-2/12 cell line (CLU177) are typically maintained in the high glucose DMEM (Gibco, 1129855) supplemented with 10% Fetal Bovine Serum (Life Technologies, 10500–064) in the presence of penicillin, streptomycin, L-Glutamine and amphotericin B (10378016, Gibco). SH-SY5Y, mHpoA-2/12 and mHippoE-14 cells were transfected with either pCDNA3 and pCDNA3-mPea3, pCDNA3-Erm or pCDNA3-Er81 (courtesy of Prof. A.D. Sharrocks) using the PEI reagent (Polysciences), in 3 replicas per sample.

### Microarray and data analysis

For microarray analysis, SH-SY5Y and mHypoA-2/12 cells were transfected as described above, and 48 h after transfection RNA samples were isolated using Absolutely RNA isolation kit (#400800, Agilent), checked for quality using Agilent Bioanalyzer System. The quality of RNA samples was evaluated based on RNA integrity number (RIN). RIN value of samples which was higher than 8 were chosen and converted to cDNA and labeled with Agilent Low Input Quick Amp Labeling Kit. The labeled cDNA was hybridized to Human Gene Expression Array 4 × 44 K (Agilent), which covers 45.033 genes with 3 probes per gene, containing 12 arrays per slide, and Mouse Gene Expression Array 4 × 44 K (The SurePrint G3 v2, Agilent). After hybridization, slides were scanned using NimbleScan 2.5 software and using three arrays from pCDNA3-transfected cell as reference samples, statistical analyses were performed under Bioconductor (https://bioconductor.org/) software platform (version 3.5.0) in R.

Agilent microarray data were processed using the Agilent expression array processing package (agilp, version 3.8.0)^34^ and normalized by the quantile normalization method. Microarray data of pCDNA3 (as control), Pea3, Erm, Er81, and Pea3-VP16 obtained from mHypoA-2/12 and SH-SY5Y cells were statistically analyzed to identify DEGs using Linear Models for Microarray Data (LIMMA) package (version 3.32.10)^35^. The False Discovery Rate was controlled through Benjamini-Hochberg’s method. The regulatory pattern of each gene (i.e., down- or up-regulation) was determined by fold changes. Raw data were submitted to EBI ArrayExpress, accession E-MTAB-8473 and E-MTAB-8475.

The discrimination performance of the differential expression levels in each condition was verified through the clustering of cells using Principal Component Analysis. The first two principal components explaining at least 80% of total variance were considered in the determination of the performance.

The gene set enrichment analysis was carried out for all DEGs through DAVID^36^ and ConsensusPathDB^37^ annotation tools. In the enrichment analyses, the KEGG^38^, Reactome^39^, and Biocarta^40^ were preferably used as the pathway databases. GO terminology^41^ was employed as the source for annotating the molecular functions and biological processes. P-values were obtained via Fisher’s Exact Test. Benjamini-Hochberg’s correction was used as the multiple testing correction technique, and gene set enrichment results with adjusted-p ≤ 0.05 were considered statistically significant.

### cDNA synthesis and qPCR

1 μg of each RNA was used to cDNA synthesis reaction using iScript cDNA Synthesis kit (1708896, BioRad) as per manufacturer’s instructions. Briefly, 4 μl of 5 × iScript Reaction Mix, 1 μl of iScript Reverse Transcriptase, 10 μl of RNA (100 ng/μl) and 5 μl of nuclease-free water. In thermal cycler, samples were incubated respectively at 25 °C for 5 min, 46 °C for 20 min and 95 °C for 1 min.

25 ng cDNA template in 10 µl reaction with SsoAdvanced universal SYBR Green supermix (172–5271) was used for Real-Time PCR and carried out using a StepOne Real-Time PCR detection system (forward and reverse primer sets are listed in Table 3). mRNA expression was normalized to β-actin and GAPDH expression. To evaluate whether the difference in gene expression level between control and transfected cells was significant, the efficiency-corrected delta cycle threshold (ΔCt) method was used according to the formula:

$$ {\text{relative quantity }}\left( {{\text{RQ}}} \right)_{{{\text{target}}}} = {\text{ E}}_{{{\text{target}}}}^{{{\text{Ct}}\left( {{\text{pCDNA3}}} \right) - {\text{Ct}}({\text{Pea3 members}})}} /{\text{E}}_{{{\text{housekeeping}}}}^{{{\text{Ct}}\left( {{\text{pCDNA3}}} \right) - {\text{Ct}}({\text{Pea3 members}})}} $$

**Table 3:** The list of primers used in qPCR assays.

Gene	Forward primer (5′–3′)	Reverse primer (5′–3′)
*Bdnf*	TTCCAGCATCTGTTGGGGAG	CTCACCTGGTGGAACATTGTG
*Efna2*	CTACATCTCTGCCACGCCTC	CTGGTGAAGATGGGCTCAGG
*Epha2*	TATGGCAAAGGGTGGGACCT	TCTCCTCGGTACACCCAGTT
*Epha4*	CCTTATTGGATTCCAGATCTGTTCA	ACTCACTTCCTCCCACCCTC
*Fgfr1*	GGCAGTGACACCACCTACTT	GAGCTACGGGGTTTGGTTTG
*hACTB*	ACGAAACTACCTTCAACTCC	GATCTTGATCTTCATTGTGCTGG
*hBAD1*	CTTTAAGAAGGGACTTCCTCGC	ATCCCACCAGGACTGGAAGA
*hEFNA1*	GCTACTACTACATCTCTCACAGTCC	TGCTATGTAGAACCCGCACC
*hEFNA3*	GCAACGCACAGACACTTTTGG	CATAGGGTGAGCAGGGCAAG
*hEFNA3*	CCACTCTCCCCCAGTTCACCATG	GCTAGGAGGCCAAGAACGTC
*hEFNA4*	CCTCGGCTTTGAGTTCTTACCT	GGGCTGACTCAGACTTCCTCT
*hEFNB1*	GGAGGCAGACAAACATGTCA	GAACAATGCCACCTTG
*hEFNB2*	GCAAGTTCTGCTGGATCAAC	AGGATGTTGTTCCCCGAATG
*hEFNB3*	GTGGCTTAGTCTGGGGGATCAG	GTTCTTCCCGCCCTTCTTCTCC
*hEGFR*	TCTTCGGGGAGCAGCGAT	CGTGCCTTGGCAAACTTTCT
*hEPHA1*	TGGCCTTGAACCTTATGCCA	GCCTGACAGTGACTCTGCAT
*hEPHA7*	GCTACAGCTGTCTCCAGTGA	CCACAGTGCCTTCTCCCAAT
*hEPHB2*	CAGACCATGACAGAAGCCGA	CACACGATGGCGATGACAAC
*hEPHB6*	GTCCCCGGACTGGAGAAGA	CCCTTTATTTCTTCCCGTTGGC
*hETS1*	GTCATTCCTGCTGCTGCCCTA	TTCCCAGCCATCTCCTGTCCAG
*hETV1*	CCCTCCATCGCAGTCCATACC	CTTGGCATCGTCGGCAAAGG
*hETV4*	CAGGCGGAGGTTGAAGAAAGG	AAGGGCAGAAGAAAGGCAAAGG
*hETV5*	TTTGATCTTGGTTGGAGGTGGGG	CTGATGATGAACAGTTTGTCCCAG
*hETV7*	TGGGAAGACAAGGACGCCAAG	GCAGGGCACGAGACATCTTC
*hFGFR1*	GTACATGATGATGCGGGACTGCTG	GAGAAGACGGAATCCTCCCCTGAG
*hGAPDH*	CATCTTCCAGGAGCGAGATCC	AAATGAGCCCCAGCCTTCTCC
*hGDNF*	TGGGAGGGGAAGGGATTAGG	GCGGCACCATTGCTGTTAG
*hGSK3B*	GGAACTCCAACAAGGGAGCA	GTTCCTGACGAATCCTTAGTCCA
*hIRS1*	CGCCGCTCAAGTGAGGATTTA	AGGTCTTCATTCTGCTGTGATG
*hL1CAM*	GCTGGTTCATCGGCTTTGTG	GTCTCATCTTTCATCGGTCGG
*hMAPK3*	TCCTGACGGAGTATGTGGCTAC	GTTAGAGAGCATCTCAGCCAGA
*hNGFR*	GAGAAAAACTCCACAGCGACAGTG	GGTAAAGGAGTCTATGTGCTCGG
*hNRCAM*	ACAACTGTGGATGAAGCTGGT	ACCAATGAACCAGCCCTGAG
*hNRG1*	GGGATTGAATTTATGGAGGCGG	GTAGGCCACCACACACATGA
*hNTNG1*	TGCCCTGCTGTGATTTGAGG	CAAGGTCCCCTCTTTGCTGG
*hNTRK3*	GCTTCGGGGTGATCCTCTGG	GCCGCTCCAAAACACGACCT
*hPIWIL4*	GGATAATTGTGTACCGTGCTGG	CTGACCACAATCACCGACAGTC
*hPLCB3*	CCCCTTCACTGAGGTCATCGT	CGTAGATGCCCACCTTCCTG
*hPLCB4*	TGGACCATCCTCTGGCTCAC	AAGACTTCCCGCCGAACTGT
*hPLXNB2*	GCAGCGTGAAAGAGAAGGAGC	CAGTGTGCCCTTGACTGAGAG
*hPRKACA*	CCAGCAGGGCTACATTCAGG	GCTCAGGATAATCTCAGGGGC
*hPTK2B*	GATGACCTGGTGTACCTCAATG	GTGTGAAGCCGTCAGCATCTG
*hROCK2*	CGCCAGAGGAAGCTGGAG	GAATTTAAGCCATCCAGCAAGC
*hSEMA3A*	GTTTTTCGGGAACCGACTGC	TGTAAAGGGAGCTGGGCAAC
*hSEMA3C*	TAATGGGCCTTTTGCCCACA	GCTCCTCCTGGACAAGTTCC
*hSEMA3D*	ACTCGATCCCTTGGGCCTAC	TTTGCTCCATTGAGCCAGTAGT
*hSEMA3E*	TTCTTCAAAGCGGCAACAGC	GCAGTCAGCACAAGCACTTC
*hSEMA4C*	CTGAGAGGACCTTGGTGTACC	GGTGAAGCCGAGTTGGAGAAG
*hSEMA5A*	GGAGGAGAGCCTGAGCATGA	CACGGAGACCACACACCAAA
*hSEMA5B*	TGTCCTGTGCGGAATGTGAC	CCTGAGTTGTCCCCATCCAA
*hSEMA6C*	ATCATAGGGCTGGAGCTGGA	AAACAGCTCCTCTGACAGGC
*hSEMA6D*	AGTCAATTTTGCTGAGCCCCT	GCCACTGAGCTACCTTCCTC
*hSEMA7A*	GCACGGACTGCGAGAACTA	ACAGTGCCATTCACCAGGTT
*hSHC2*	CATGCCGTCCATCTCCTTCG	CAGGATGTGGCAGGCTCTCT
*hTP53*	CTACCAGGGCAGCTACGGTT	TTGTTGAGGGCAGGGGAGTA
*hWNT5A*	CCAGGCTTAACCCGGTCG	CAATGGACTTCTTCATGGCGAG
*mActnb*	CTTGGGTATGGAATCCTGTGG	TGGCATAGAGGTCTTTACGG
*mBad*	CTTCAAGGGACTTCCTCGCC	CCCAAGTTTCGATCCCACCA
*mEfna1*	CCTCTCTTGGGTCTGTGCTG	GTCCTCCTCACGGAACTTGG
*mEfna4*	CCCTTTCAGCCCTGTTCGAT	GAGTCGGCACCGAGATGTAG
*mEfnb3*	GCTGCTGTTAGGTTTTGCGG	CCTGGAACCTCTTATTCGCCG
*mEgfr*	TGGGTGGCCTCCTCTTCATA	AGGTTCCACGAGCTCTCTCT
*mEpha1*	TGTGGACCTCCAGGCCTAT	CACCAAACTCCCCTTCTCCTATG
*mEpha7*	AACCGGGAACAGTGTACGTC	ACTGCTGTCGCTTCAAACAT
*mEphb2*	AAGCTACCCCTCATCGTTGG	CTCAAACCCCCGTCTGTTACAT
*mEphb6*	CTACCAGACAAGAAGGGAAGCAAG	CATGCGGTCTCTTCGGCA
*mEr81*	GGGGAAGTGCTGGGCAATAA	TATCAACCTGAAGGCCACGC
*mErm*	CTGGAAGGCAAAGTCAAGCAG	TAAATTCCATGCCTCGGCCA
*mGapdh*	CATGACCACAGTCCATGCCATC	ACGGACACATTGGGGGTAGG
*mGdnf*	ACCAGTGACTCCAATATGCCTG	CTGCCGCTTGTTTATCTGGTG
*mGsk3b*	ACCGAGAACCACCTCCTTTG	TGCTGCCATCTTTATCTCTGCT
*mIRS1*	TTAGGCAGCAATGAGGGCAA	TCTTCATTCTGCTGTGATGTCCA
*mL1cam*	AGTCCAGGCAGTGAACAACC	GCTCACCTGGGGGTAGTCTT
*mNrcam*	ATGCACAGACATCAGTGGGG	GTGGAGGAATACCAGCTTCGT
*mNrg1*	GAGTGCAGACCCATCTCTCG	CCAGGGCTTCTCCCATCTTC
*mNtf3*	GGTAGCCAATAGAACCTCACCAC	GTCACACACTGAGTACTCTCCTC
*mPea3*	GCTCGCAGAAGCTCAGGTA	GGTGGTGGGGCTATGGAAAG
*mRock2*	TCAGAGGAAGCTGGAGGCG	AGGAATTTAAGCCATCCAGCAGA
*mSema3a*	TACTGCAAAGAGGCGCACAA	GGCTGGGCCCATGATGATTA
*mSema3c*	CCCACCTCGGTATTTTCCCTT	AACACACAAATCGCCCGGAA
*mSema3d*	TGGGAAAGATGCCAATGCAGA	GTACCCACACAGCGGATGAA
*mSema3e*	TGTCCACGCTAGTTGGGAAT	ATGGCCTAACTTCCCCATGC
*mSema6c*	GCTTAGGGAGGGTGCAGTTT	CCAGGGACAGAGCAGTTGAG
*mSema6d*	TTTGTATGAGCCGCGTCTTT	ACTTAGCTACCTGGTTGTTGGG
*mSema7a*	CTTCTGCTGGTGTTCTGGGT	CATGGTCCTGCCCTTTCCAG
*mWnt5a*	CAGCCCTGCTTTGGATTGTC	AATGGGCTTCTTCATGGCGAG
*Sema3f*	TGCCTGGTCAACAAGTGGAG	TGGACAAACACGTCCTGGAG
*Sema3g*	CGTCTGCGTGAATGATGCTG	CTGGTCAAAGTGGGTCTCGG
*Sema4f*	AGTGCGGGGTTATTGATGTGT	AGCCATTACAGCTGCTGACC
*Sema5b*	GTGCAGCAACAACTGTGGAG	CAGGTCTTGAACTCCACGCC
*Spred1*	GGACTAAGCAGCGTCACTGT	TCCAAAACCACCATTTTGTCCC
*Src*	GGTGGAGTGACCACCTTTGT	CCAGTCTCCCTCTGTGTTGT
*Unc5c*	CGGACTGGGACTGGGATACT	AGTCATCATCTTGGGCGGC
*Vegfa*	GCTCAGAGCGGAGAAAGCAT	GTCACATCTGCAAGTACGTTCG

Primers for human genes, denoted in all capital letters, were used for validation in human SH-SY5Y cells, whereas primers specific for mouse genes, denoted with only first letter capitalized, is used for validation in mouse mHypo-A2/12 and mHippo-E14 cells.

The RQ values thus calculated were then transformed on a log2 scale to achieve normal distribution of the data and the resulting distributions were tested against the null-hypothesis of equal mRNA level in control and transfected cells (i.e., a population mean of 0.0) using two-tailed one-sample Student’s t-tests. A confidence level of α ≤ 0.05 was applied for all comparisons to determine statistical significance. After transfection of Pea3, Erm and Er81 into cells, qPCR was used to determine overexpression levels in transfected cells, and it was observed that the fold changes of Pea3, Erm and Er81 in each cell type was not significantly different except for SH-SY5Y cells, where Erm expression was slightly less than Pea3 or Er81 in transfected cells (Supplemental Fig. S1).

### Analysis of the promoter sequences for transcription factor binding

The promoter sequences were obtained from Transcriptional Regulatory Element Database of Cold Spring Harbor (TRED) (https://rulai.cshl.edu/cgi-bin/TRED/tred.cgi?process=home) and Eukaryotic Promoter Database (EPD) (https://epd.vital-it.ch/search_EPDnew.php), and analyzed with PROMO 3.0 to determine the putative binding site sequences of Pea3 on the promoter sequences and their dissimilarity rates, which indicate the variance between DNA-binding sequence of transcription factor and the nucleotide sequence on the selected promoter as percentage by regarding the binding matrices^42^. Thus, the higher possibility for Pea3 binding is stated as the smaller dissimilarity rates (0% dissimilarity rate indicates 100% identity to the consensus motif). Furthermore, genomic sequences are scanned and used to convert into Position Weight Matrices (PWMs) via JASPAR (https://jaspar.genereg.net/) to determine the binding score and their statistical significance (For evaluation, the binding score was taken to be p < 0.01 (JASPAR) and ≤ 13% (PROMO).

### Chromatin immunoprecipitation

Chromatin immunoprecipitation was performed according to a protocol described previously^13^ with some modifications. Briefly, SH-SY5Y neuroblastoma cells were transfected with either empty pCMV-6-tag flag or pCMV-Pea3-flag expression plasmid. 48 h after transfection, cells were cross-linked with 1% formaldehyde, and then lysed in lysis buffer. For chromatin shearing, the lysates were sonicated using Bioruptor Pico sonication device (Diagenode) in nuclei isolation buffer. 10% v/v of the sheared DNA was saved as input. The rest of samples was precipitated with anti-Flag M2 affinity resin (Sigma, A2220) or normal mouse IgG (Santa Cruz, sc-2025) overnight. After overnight incubation, immunoprecipitated chromatins were eluted in elution buffer. Crosslinking of DNA and protein was reversed with heat treatment in high salt solution, and then treated with RNase and proteinase K. DNA in the samples was purified using MEGAquick-spinTM Total Fragment DNA Purification Kit (Intron). Purified DNAs (input and ChIP samples) were detected by qPCR using SsoAdvanced universal SYBR Green supermix (BioRad). MMP2 and MMP9 promoter regions were used as positive controls, and FGFR1 intron region as a negative control. Primers are listed in Table 4. ChIP-qPCR data was analyzed as described previously^13^.

**Table 4:** The list of primers used in chromatin immunoprecipitation qPCR assay in SH-SY5Y cells.

Gene	Forward primer (5′–3′)	Reverse primer (5′–3′)
*hMMP9*	TAAGACATTTGCCCGAGGTC	CCTCTTTTTCCCTCCCTGAC
*hNEGATIVE*	GGACGTGGAGGGCTAGGTTA	TTAACGACCGTGGGTTGTCC
*hSEMA5A*	CCGCAACGACTGACTCTCTC	GCGGTTCCCTCGGCTAAATC
*hBAD1-1*	CAGGCGGAGCGGTAACAATAG	TGTGAAGGGAGTGATTACTGCGG
*hBAD1-2*	GACCCCAGAGAATCCCTCTCCT	CTTTTTCCTCCCTCCTTCGGC
*hEFNA3*	AGGCGAATGGGTTGTCTTGG	ACACACCGACATCTCTCTGC
*hETS1*	GAGGACACGGGCTCACGAAT	CAGATCCTGAGTGAGCTTCCC
*hSEMA5B*	TCTGGGTCCTGCCCTAGAAA	CACGCTACCTGGCTGGTC
*hEPHB3*	GGGGGACTTTGAAGAAATACTGCC	CTCTGGGCAGGGGTGACATT
*hEPHA7*	CGAAAGCTGCGGTTGCTAAAAGC	GTTGCTGGTCCAGGATTCCCTC
*hSEMA3A*	GACACTCAGCAGGGACTTTTGTG	GGAATGCTCTGGCTAACATTTTC
*hSEMA3E*	ACGTGGACCAGAAATACTTCCTAG	TTCCCTCTAGGAGTCGCAGGATC

### Visualization of protein structures

The structures of Etv1, Etv4, and Etv5 bound with DNA were compared as visualizing via Visual Molecular Dynamics (VMD), molecular visualization program, (Version 1.9.3; https://www.ks.uiuc.edu/Research/vmd/)^43^. The structures of Etv1, Etv4 and Etv5 were obtained from the PDB database with PDB IDs of 4BNC, 4UUV, and 4UNO, respectively. The structures of Etv1 and Etv5 without DNA were aligned to the structure of Etv4 with DNA. Each different residue of these three proteins was colored by blue, red and green for Etv1, Etv4, and Etv5, respectively. The different residues were 334-Ser, 343-Ala, 349-Ser, 374-Arg, 417-Asp of Etv1; 340-Ala, 349-Ala, 355-Thr, 380-Leu, 423-Glu of Etv4; and 367-Ser, 376-Thr, 382-Ala, 407-Arg, 450-Asp of Etv5.

## Supplementary information

Supplementary Information 1.

Supplementary Information 2.
