# Supplementary figures and images for: Transcriptomic profile of Pea3 family members reveal regulatory codes for axon outgrowth and neuronal connection specificity
Source: Sci Rep. 2020 Oct 23;10:18162. doi: 10.1038/s41598-020-75089-3 (PMC7584614; doi:10.1038/s41598-020-75089-3)

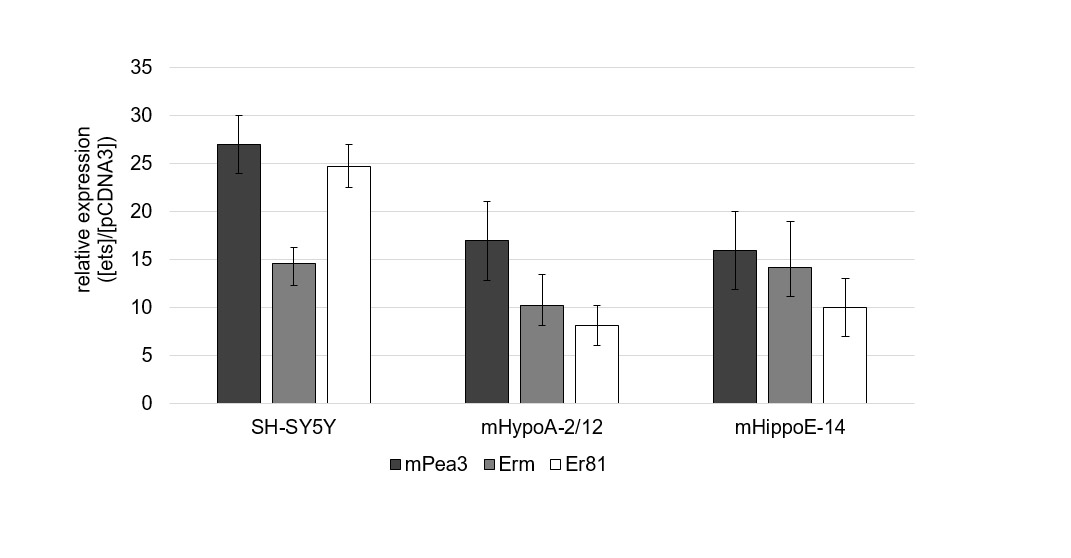

Supplement: Supplementary file 1 — Supplementary Information 1. [file 41598_2020_75089_MOESM1_ESM.jpg]
