# Supplementary material for: Transcriptomic profile of Pea3 family members reveal regulatory codes for axon outgrowth and neuronal connection specificity
Source: Sci Rep. 2020 Oct 23;10:18162. doi: 10.1038/s41598-020-75089-3 (PMC7584614; doi:10.1038/s41598-020-75089-3)
Supplement: Supplementary file 2 — Supplementary Information 2. [file 41598_2020_75089_MOESM2_ESM.docx]

**Transcriptomic profile of Pea3 family members reveal regulatory codes for axon outgrowth and neuronal connection specificity**

Başak Kandemir^1,2ξ^, Gizem Gülfidan^3^, Kazim Yalcin Arga^3^, Bayram Yilmaz^2,4^, and Isil Aksan Kurnaz^1,5*^

^1^Gebze Technical University, Institute of Biotechnology, Kocaeli, Turkey

^2^Yeditepe University, Biotechnology Graduate Program, Kayisdagi, Istanbul, Turkey

^3^Marmara University, Department of Bioengineering, Goztepe, Istanbul, Turkey

^4^Yeditepe University, Faculty of Medicine, Kayisdagi, Istanbul, Turkey

^5^Gebze Technical University, Department of Molecular Biology and Genetics, Kocaeli, Turkey

^ξ^ Present address: Baskent University, Department of Molecular Biology and Genetics, Ankara

*Corresponding author: Isil Aksan Kurnaz; [ikurnaz@gtu.edu.tr](mailto:ikurnaz@gtu.edu.tr)

**Supplementary Figure 1 Legend.**

Relative expression levels of exogenous Pea3, Erm and Er81 in SH-SY5Y, mHypoA-2/12 and mHippoE-14 cells transfected with appropriate expression plasmids. qPCR results were normalized to that of pCDNA3-transfected cells in each case, and statistical analysis was done as described in Materials and Methods. No significant different in expression was determined in mHypoA-2/12 and mHippoE-14 cells, while in SH-SY5Y cells exogenous Erm expression was almost 0.5-fold of those of Pea3 or Er81. Values are expressed as mean ± SEM. *p* values: * < 0.05; ** < 0.01; *** < 0.001; Student’s t-test.

**Supplementary Table S1.** Nervous system-related pathways that were chosen for further analysis among enriched pathways in Pea3-, Erm-, and Er81-transfected mHypoA-2/12 cells. GO: Gene ontology, R-HAS: Reactome, path:has: KEGG term.

|  | **Term** | **Description** | **p-Value** | **Times found** | **Gene Symbol in Enrichment results -Down** |
| --- | --- | --- | --- | --- | --- |
| **PEA3** | GO:0022008 | Neurogenesis | 2,43E-03 | 401 | NRCAM; TBX20; IST1; B2M; CCK; SEMA4A; BDNF; LEMD2; AXL; TOPORS; OGDH; USP33; RAPH1; CCDC88A; SECISBP2; MIB1; RORA; PARK2; LIG4; INHBA; CSNK1E; GOLGA4; NAPA; RELA; KNDC1; SEMA7A; NCKIPSD; LRRC4C; CREB1; EIF2B3; CLCF1; LDB1; IFRD1; EEF2; CXCL12; PAFAH1B1; FGFR1; KCTD11; NME1; BRSK1; KIF5C; CSF1; CEP290; RUNX1; ENC1; GRIP1; WNT7A; ADCY1; CCL2; STMN4; NGF; CCDC66; VLDLR; CCL5; COPS2; GORASP1; KIF26B; MED1; SMARCE1; LAMB1; NDRG1; VANGL2; TNFRSF12A; ARNTL; APOE; CHD5; EGR2; NBL1; TULP1; JUN; OTX2; RTN4; NGRN; TBC1D24; TOP2B; DLX2; PDLIM5; CYB5D2; DPYSL3; UNC5C; UBA6; PPARG; NFASC; MAP1S; VEGFA; VEGFC; GRN; LAMC3; ZEB1; ZEB2; CDK5RAP1; ETV5; ETV4; CUX1; ELP3; NOG; WDR36; ALKBH1; EFNB3; NUMB; EPHA7; ONECUT2; EIF2B2; EPHA2; VAPA; KIF17; TBX6; ABL2; ADNP; AREG; NCOA1; MARCKS; RANBP9; S100A8; BHLHB9; LAMA2; COL25A1; CELSR3; KIDINS220; WHRN; PARD3; ARFGEF1; MOB2; UBE2V2; MAPK8IP3; LRRC38; GPR37L1; ZFYVE27; SGK1; MICALL1; STX3; SMURF1; ATL1; IL6; COBL; FOXP1; CDK5RAP2; RAPGEF2; RHEB; SPG11; NRN1L; CAMSAP1; DHFR; BCL11A; LGI4; ARHGAP4; EZH2; MAP6; WDR81; IMPACT; ANKRD1; CRABP2; RND1; PHLDA1; UHMK1; MED12; SYNJ1; SOCS7; NRG1; ADNP2; SPINK5; JAG1; YWHAE; ITGB1; CNTF; NEPRO; MTR; HMGA2; REST; RRN3; SKIL; TNC; NDEL1; MCOLN3; PRMT5; AGTPBP1; DUSP10; MUL1; HAP1; AXIN1; NCK2; RTN4R; ATF1; FEZ2; BBS4; IER2; NEDD4L; MBD1; GDNF; RDH13; WNT16; BCL6; PTPRK; WNT11; PCSK9; IFT20; RYK; STK11; OGN; LRFN3; FAIM2; AKT2; BOK; CPNE5; KIRREL3; NKX2-2; CIB1; USP21; GSN; EEF2K; LINGO4; ADAMTS1; BLOC1S1; CNGB1; CACNA1A; EML1; ADRA2B; NTRK3; NAV1; FMOD; CDK5R1; ERBB2; CDKN2C; DDIT4; SHC1; UBE4B; WNK1; CAMK2B; TLE6; LLGL2; INPP5E; TRAPPC9; STXBP1; BMP4; LST1; MTCH1; HIPK2; PACSIN1; APP; SEMA3F; NRXN3; PTK2B; GSK3B; RUNX2; PTEN; DDX6; GRB2; FKBP1B; MAG; TCF12; PRRX1; SNAPIN; LRP8; PAK3; EVL; STMN1; THOC2; HRAS; DCLK1; KAT2B; NDNF; COL3A1; DAGLB; PTN; APOD; PREX2; TULP3; TCTN1; SOX14; CDON; ITPKA; JAK2; CX3CL1; GBA2; DLX1; NRTN; EPHB2; TCF4; DPYSL2; EFEMP1; DIXDC1; TUBB3; MAN2A1; VEGFD; LRFN1; DAG1; CDK5RAP3; RHOA; PTPN5; EPOR; PMP22; SH3TC2; SDC2; SDC4; BARHL1; B4GAT1; FBXO31; FLOT1; WNT10A; BAIAP2; AIFM1; MYH10; HDAC5; NRN1; HDAC9; SPHK1; SMO; OLFM1; MBOAT7; CTNND2; NFATC4; NR2F2; CD9; GPRC5B; FZD2; FZD8; ASPM; ARF1; NR2F6; CBLN1; HNRNPK; NRP2; RAB10; MAPK9; ATAT1; RIMS1; NTF3; ROM1; MECP2; EFNA4; EFNA3; EFNA2; EFNA1; FOXD1; OPRM1; MTOR; FES; CLU; FRY; ADM; TSPO; SEMA3A; CNTNAP1; SEMA3C; LRP6; SEMA3D; OMD; WNT1; MICALL2; ID2; WNT6; ID1; SPP1; CAMSAP3; SEMA3E; KIF20B; ASAP1; CDH11; TGFB2; IL1RAPL1; HMGB1; LIMK1; ROGDI; CTNNB1; SOX9; NDE1; ZMYND8; SEMA6C; NPTN; TWIST1; FAS; SLC9A3R1; NRAS; RB1; RSPO2; SLIT2; SIPA1L1; MBP; USH1G; NUMBL; ULK2; POU3F1; MATN2; CNP; RAC1; HOOK3; PIGT; RAB3A; CTNNA2; NFIB; ARX; CALR; CALU; NOTCH3; PTPRF; GSTP1; CUL4B; MAPT; SERPINF1; MYO7A; SLC1A3 |
|  |  |  |  |  |  |
|  | GO:0030182 | Neuron differentiation | 7,21E-03 | 348 | NRCAM; TBX20; IST1; B2M; CCK; SEMA4A; BDNF; TOPORS; OGDH; USP33; RAPH1; PAFAH1B1; MIB1; RORA; PARK2; INHBA; CSNK1E; GOLGA4; NAPA; KNDC1; NCKIPSD; LRRC4C; CREB1; LDB1; IFRD1; CXCL12; SEMA7A; FGFR1; KCTD11; NME1; BRSK1; KIF5C; CEP290; RUNX1; ENC1; GRIP1; WNT7A; ADCY1; STMN4; NGF; CCDC66; VLDLR; CCL5; COPS2; GORASP1; KIF26B; MED1; WHRN; LAMB1; VANGL2; TNFRSF12A; SMURF1; APOE; CHD5; EGR2; NBL1; TULP1; JUN; OTX2; RTN4; NGRN; TBC1D24; TOP2B; DLX2; PDLIM5; DPYSL3; UNC5C; UBA6; CUX1; NFASC; MAP1S; VEGFA; GRN; CYB5D2; ZEB1; ZEB2; ETV5; ETV4; NOG; WDR36; CCDC88A; ALKBH1; EFNB3; NUMB; EPHA7; ONECUT2; RTN4R; EPHA2; VAPA; FOXP1; TBX6; ABL2; ADNP; AREG; NCOA1; MARCKS; RANBP9; BHLHB9; LAMA2; COL25A1; CELSR3; KIDINS220; PARD3; ARFGEF1; MOB2; UBE2V2; MAPK8IP3; LRRC38; GPR37L1; ZFYVE27; SGK1; MICALL1; STX3; ATL1; IL6; COBL; CDK5RAP2; RAPGEF2; CDK5RAP1; SPG11; NRN1L; CAMSAP1; DHFR; BCL11A; LGI4; ARHGAP4; EZH2; MAP6; IMPACT; ANKRD1; AXIN1; RND1; PHLDA1; UHMK1; NRG1; ADNP2; SPINK5; JAG1; SECISBP2; ITGB1; CNTF; NEPRO; MTR; REST; RRN3; SKIL; TNC; NDEL1; MCOLN3; AGTPBP1; MUL1; CRABP2; NCK2; ATF1; FEZ2; BBS4; IER2; NEDD4L; MBD1; GDNF; RDH13; WNT16; BCL6; PTPRK; WNT11; PCSK9; IFT20; RYK; STK11; OGN; LRFN3; HIPK2; PTK2B; CPNE5; KIRREL3; NKX2-2; CIB1; USP21; EEF2K; LINGO4; ADAMTS1; BLOC1S1; CNGB1; CACNA1A; ADRA2B; NTRK3; FMOD; CDK5R1; ERBB2; FAIM2; DDIT4; SHC1; UBE4B; WNK1; CAMK2B; TLE6; SEMA3E; INPP5E; TRAPPC9; STXBP1; BMP4; LST1; MTCH1; PACSIN1; APP; SEMA3F; NRXN3; LRFN1; GSK3B; RUNX2; PTEN; DDX6; GRB2; FKBP1B; MAG; TCF12; PRRX1; SNAPIN; LRP8; PAK3; EVL; STMN1; THOC2; HRAS; DCLK1; KAT2B; NDNF; PTN; APOD; PREX2; TULP3; TCTN1; CDON; ITPKA; JAK2; GBA2; DLX1; NRTN; EPHB2; TCF4; DPYSL2; EFEMP1; DIXDC1; TUBB3; VEGFD; DAG1; RHOA; PTPN5; EPOR; PMP22; CAMSAP3; SDC2; SDC4; B4GAT1; FBXO31; FLOT1; WNT10A; BAIAP2; AIFM1; MYH10; HDAC5; NRN1; HDAC9; SPHK1; SMO; OLFM1; CTNND2; NFATC4; GPRC5B; FZD2; FZD8; ASPM; ARF1; NR2F6; CBLN1; HNRNPK; NRP2; RAB10; MAPK9; ATAT1; RIMS1; NTF3; ROM1; MECP2; EFNA4; EFNA3; EFNA2; EFNA1; FOXD1; MTOR; FES; CLU; FRY; ADM; TSPO; SEMA3A; CNTNAP1; SEMA3C; LRP6; SEMA3D; OMD; WNT1; MICALL2; ID2; WNT6; ID1; SPP1; CDK5RAP3; KIF20B; ASAP1; CDH11; TGFB2; IL1RAPL1; HMGB1; LIMK1; CTNNB1; SOX9; ZMYND8; SEMA6C; NPTN; FAS; SLC9A3R1; NRAS; RB1; RSPO2; SLIT2; SIPA1L1; MBP; USH1G; NUMBL; ULK2; MATN2; CNP; RAC1; PIGT; RAB3A; CTNNA2; NFIB; ARX; CALR; CALU; NOTCH3; PTPRF; CUL4B; MAPT; SERPINF1; MYO7A; SLC1A3 |
|  | path:hsa04722 | Neurotrophin signaling pathway | 5,95E-03 | 46 | NGF; MAP2K5; CRKL; JUN; NFKBIA; FOXO3; PLCG2; AKT3; MAPK12; CRK; BDNF; TRAF6; PIK3CD; PIK3R5; RELA; YWHAE; SHC2; CAMK2D; KIDINS220; CAMK2A; SH2B2; RPS6KA3; PRDM4; ATF4; HRAS; MAPK14; BAD; CALM2; CALM3; CALM1; PIK3CA; NRAS; NTRK3; PIK3R3; PIK3R2; MAPK9; NTF3; SHC1; RAC1; CAMK2B; SH2B3; RHOA; MAGED1; AKT2; GSK3B; GRB2 |
|  | GO:0048675 | Axon extension | 2,17E-02 | 41 | ITGB1; SEMA4A; APOE; RTN4; ADNP; RAPH1; NRCAM; SEMA7A; BCL11A; NRG1; TNFRSF12A; WDR36; GOLGA4; IFRD1; NDEL1; ARHGAP4; VEGFA; CXCL12; PAFAH1B1; RTN4R; SPG11; ULK2; SEMA3A; SEMA3C; DPYSL2; FMOD; SEMA3F; LIMK1; DCLK1; NTRK3; OLFM1; OGN; CDK5R1; SLIT2; MAPT; SEMA3D; SEMA3E; GSK3B; SEMA6C; NRP2; RYK |
|  | GO:0061564 | Axon development | 1,36E-03 | 72 | CDH11; ERBB2; TGFB2; STMN1; RYK; HRAS; LIMK1; DCLK1; CDK5R1; MYH10; STK11; OLFM1; LRFN3; EVL; SEMA6C; APOD; LINGO4; PTPRF; CACNA1A; TCTN1; NRAS; NTRK3; OGN; JAK2; NRP2; FMOD; TUBB3; SIPA1L1; SPP1; MBP; STXBP1; NUMBL; EPHB2; B4GAT1; MATN2; DPYSL2; MAPT; CNP; RAC1; SLIT2; EFNA4; EFNA3; EFNA2; EFNA1; FOXD1; LRFN1; DAG1; RAB3A; RAB10; RHOA; TSPO; CTNNA2; SEMA3A; NFIB; SEMA3C; SEMA3E; SEMA3D; OMD; ARX; APP; SEMA3F; NRXN3; CALU; GSK3B; PTEN; ULK2; GRB2; FKBP1B; FLOT1; MAG; BAIAP2; PAK3 |
|  | path:hsa04360 | Axon guidance | 1,30E-03 | 35 | NFATC4; RYK; PPP3R1; HRAS; LIMK1; SRGAP3; SMO; EFNA1; DPYSL2; SEMA6C; PIK3CA; NRAS; PIK3R3; PIK3R2; SLIT2; EPHB2; EPHB6; EPHB4; RAC1; EFNA4; EFNA3; EFNA2; CAMK2B; FES; RHOA; SEMA3D; SEMA3A; SEMA3C; SEMA3E; NCK1; SEMA3F; GSK3B; PDK1; EPHA1; PAK3 |
|  | R-HSA-422475 | Axon guidance | 3,72E-03 | 77 | MYH10; PDGFRB; PDGFRA; EVL; HRAS; LIMK1; EGFR; ITGB3; SPRED2; CNTNAP1; ARAF; ARRB1; EPHB6; GSK3B; RGMB; SPRED1; SHC1; CACNA1I; CALM2; CALM3; CALM1; CACNB3; NRAS; PFN2; CACNA1G; SRGAP3; FGF18; JAK2; NRP2; KSR1; NRG2; NRTN; SLIT2; EPHB2; IL5RA; CDK5R1; DPYSL2; ANGPT1; EPHB4; RAC1; ARPC3; EFNA4; EFNA3; RDX; CAMK2B; EFNA2; COL4A5; COL4A4; IL3RA; EFNA1; FGF7; SEMA3E; RHOA; LAMC1; CSNK2A1; MYL12B; SEMA3A; EPHA1; DUSP6; RPS6KA4; NCK1; ERBB2; ARPC1A; AGAP2; SCN3A; GFRA4; ANK2; GRB2; DOCK1; FES; GFRA2; COL6A1; DNM1; COL6A2; SDC2; LAMTOR2; PAK3 |
|  | GO:0007411 | Axon guidance | 2,00E-02 | 35 | MYH10; ERBB2; TGFB2; EVL; RYK; HRAS; SEMA6C; NRAS; OGN; CDK5R1; SLIT2; FMOD; EPHB2; MATN2; DPYSL2; RAC1; EFNA4; EFNA3; EFNA2; EFNA1; FOXD1; DAG1; NRP2; SEMA3A; NFIB; SEMA3C; SEMA3E; SEMA3D; SEMA3F; ARX; APP; NRXN3; B4GAT1; TUBB3; GRB2 |
|  | GO:0031103 | Axon regeneration | 4,19E-02 | 9 | APOD; PTPRF; SPP1; CALU; NTRK3; PTEN; FKBP1B; JAK2; TSPO |
| **Erm** | GO:0022008 | Neurogenesis | 4,40E-05 | 98 | DYNLL2; FKBP4; B2M; BOK; GSN; LINGO4; PPT1; ZSWIM6; THY1; CDH2; MTMR2; ARHGEF10; CDKN2C; NGEF; SHC1; WDR1; WNK1; TSPAN2; PER2; SEMA3E; USP33; PAFAH1B1; UBE4B; CSPG4; APP; GSK3B; ENC1; PRRX1; SNAPIN; CCDC66; STMN1; VLDLR; PDLIM5; ATP7A; CDK1; TCF4; DPYSL2; DPYSL3; UNC5C; MAN2A1; CUX1; DAG1; CYB5D2; ETV5; PPARG; SDC2; MEF2C; ASPM; NAB2; NRN1; EPHA7; ILK; VAPA; SMO; FABP7; CTNND2; EVL; NCAM1; FZD1; FZD2; ALCAM; NR2F6; ARF4; FBXW8; CIB1; TMEM30A; BHLHB9; ATAT1; BEND6; LAMA2; RAP2A; SERPINF1; CLU; WEE1; DICER1; ID2; ARX; KIF20B; HMGB1; NCS1; PTPRZ1; MAP2; NDE1; NTF3; FMR1; IQGAP1; NRAS; UHMK1; SPINK5; YWHAE; TMEM106B; VCL; LSM1; NRP1; PBX1; VAMP7; FEZ1; ATF5 |
|  | GO:0030182 | Neuron differentiation | 1,69E-04 | 84 | CCDC66; STMN1; VLDLR; PDLIM5; EPHA7; HMGB1; ILK; VAPA; PTPRZ1; FKBP4; B2M; RAP2A; CTNND2; ASPM; FMR1; EVL; ATP7A; NCAM1; SHC1; NRN1; IQGAP1; FZD2; LINGO4; DYNLL2; ALCAM; NR2F6; NRAS; UHMK1; SMO; ARF4; FBXW8; ZSWIM6; CIB1; CYB5D2; TMEM30A; DICER1; THY1; CDH2; ATAT1; MTMR2; FZD1; SNAPIN; BEND6; TCF4; LAMA2; NTF3; DPYSL2; DPYSL3; UBE4B; UNC5C; WNK1; VCL; LSM1; NCS1; TSPAN2; CUX1; SERPINF1; DAG1; CLU; USP33; PAFAH1B1; WEE1; PBX1; NGEF; MAP2; BHLHB9; ETV5; VAMP7; ARX; TMEM106B; APP; ID2; GSK3B; MEF2C; FEZ1; ATF5; ENC1; PPT1; PRRX1; SEMA3E; NRP1; KIF20B; SDC2; SPINK5 |
|  | R-HSA-422475 | Axon guidance | 9,80E-06 | 47 | EVL; EPHA7; PRNP; SPRED1; SRGAP3; NGEF; RGMB; IQGAP1; MYL9; KBTBD7; CALM2; CALM1; ALCAM; MYH9; NRAS; NCAM1; ANGPT1; DUSP1; PDGFA; DPYSL2; DPYSL3; PRKCA; UNC5C; VCL; ARPC5; COL4A5; COL4A4; COL4A3; LAMC1; NRP1; RPS6KA2; RPS6KA3; SEMA3E; MYL12B; MYL12A; SDC2; IRS1; ROCK2; GSK3B; PRKACB; COL6A1; COL6A3; COL6A2; ACTR3; SCN3A; SHC1; PPP2R5C |
|  | path:hsa04722 | Neurotrophin signaling pathway | 1,36E-02 | 11 | PIK3R1; NFKBIB; NFKBIA; BAX; NFKBIE; PIK3R5; SH2B2; SH2B3; MAPK12; NFKB1; NTRK3 |
| **Er81** | GO:0022008 | Neurogenesis | 2,67E-07 | 134 | BEND6; MCOLN3; TCF4; PER2; HMGB1; EPHA4; FMN1; PTPRZ1; MAP2; FABP7; BOK; TBX6; CTNND2; GABRB1; FMR1; TMEM106B; RAP2A; ASPM; PTN; APOD; LINGO4; SEMA3D; GCM1; CNGB1; PPT1; NRN1; ZSWIM6; HOOK3; DICER1; THY1; CDH2; HAP1; ARHGEF10; CDKN2C; NTF3; COL25A1; FZD1; UNC5C; VCL; FZD2; CLU; TSPAN2; CUX1; MEG3; SATB2; RAB3A; NGEF; LRP6; FRY; SPINK5; KIF3A; PBX1; ZMYND8; FLRT1; ETV5; VAMP7; CSPG4; PPARG; ARX; LRP8; HEXB; ID2; EIF2AK4; TLX2; GSK3B; MEF2C; BMPR2; TCF12; HDAC11; SEMA3E; WNT5A; PAK3; WNT7A; NAB2; CDH11; CCL2; LIF; FOXP1; IGSF9; CCL5; DTNBP1; SPHK1; CNTNAP1; KATNA1; LAMC3; STMN4; NDRG1; SOX4; GPRC5B; MYCN; STAT3; RND1; FUOM; FAS; TCTN1; CSF1R; NTRK3; WDPCP; RIMS1; ALKBH1; EPHB2; FLOT1; SOD2; NEPRO; ULK4; BARHL1; EFNA3; KCTD11; CELSR1; PRMT5; TLE6; SKIL; VEGFC; PAX2; SLC11A2; SEMA3C; MUL1; BRSK1; LST1; SRRT; STX3; MICALL2; LRFN1; USH1G; IL6; ARTN; WDR36; GDNF; CAMSAP3; APOA5; BCL6; IL15RA; ROBO3; IFRD1 |
|  | GO:0030182 | Neuron differentiation | 1,20E-05 | 113 | MCOLN3; NRN1; HMGB1; EPHA4; FMN1; PTPRZ1; MAP2; RAP2A; TBX6; CTNND2; GABRB1; FMR1; TMEM106B; ASPM; PTN; APOD; LINGO4; CNGB1; PPT1; ZSWIM6; DICER1; THY1; CDH2; BEND6; TCF4; NTF3; COL25A1; FZD1; UNC5C; VCL; FZD2; CLU; TSPAN2; CUX1; MEG3; SATB2; RAB3A; NGEF; LRP6; FRY; SPINK5; KIF3A; PBX1; ZMYND8; FLRT1; ETV5; SEMA3D; VAMP7; ARX; LRP8; ID2; EIF2AK4; TLX2; GSK3B; MEF2C; BMPR2; TCF12; SEMA3E; WNT5A; WNT7A; PAK3; CDH11; STMN4; LIF; FOXP1; IGSF9; CCL5; DTNBP1; SPHK1; CNTNAP1; KATNA1; APOA5; SOX4; GPRC5B; MYCN; STAT3; RND1; FUOM; FAS; TCTN1; CSF1R; NTRK3; WDPCP; RIMS1; ALKBH1; EPHB2; FLOT1; SOD2; NEPRO; ULK4; EFNA3; KCTD11; TLE6; SKIL; PAX2; SLC11A2; SEMA3C; MUL1; BRSK1; LST1; CAMSAP3; STX3; MICALL2; LRFN1; USH1G; IL6; ARTN; WDR36; GDNF; BCL6; IL15RA; ROBO3; IFRD1 |
|  | R-HSA-422475 | Axon guidance | 1,95E-03 | 26 | PDGFRB; MYH14; EPHA4; SRGAP3; CALM1; MYH9; PRNP; KDR; PDGFA; ITGB3; NGEF; UNC5C; VCL; COL4A5; COL4A4; COL4A3; RPS6KA2; SEMA3E; MYL12B; PPP2CA; GSK3B; MET; COL6A3; COL6A2; SCN3A; PAK3 |
|  | path:hsa04360 | Axon guidance | 1,39E-02 | 11 | NGEF; SEMA3D; UNC5C; EPHA4; SRGAP3; GSK3B; MET; BMPR2; SEMA3E; WNT5A; PAK3 |
|  | GO:0061564 | Axon development | 3,82E-02 | 20 | MEG3; APOD; LINGO4; SEMA3E; SEMA3D; ARX; PTPRZ1; EPHA4; VCL; GSK3B; TSPAN2; BMPR2; RAB3A; UNC5C; COL25A1; THY1; CDH2; WNT7A; WNT5A; PAK3 |
|  | path:hsa04722 | Neurotrophin signaling pathway | 1,15E-02 | 10 | NFKBIB; NFKBIA; CAMK4; NFKBIE; PIK3R5; SH2B2; IRAK3; NFKB1; MAP3K5; NTRK3 |

**Supplementary Table S2.** Comparison of microarray and qPCR results in Pea3-, Erm- and Er81-transfected SH-SY5Y cells.

| **Neurotrophin signaling and development** |  | **Pea3** | | | | **Erm** | | | | | **Er81** | | | |
| --- | --- | --- | --- | --- | --- | --- | --- | --- | --- | --- | --- | --- | --- | --- |
|  |  | **Microarray** | | **qPCR** | | **Microarray** | | | **qPCR** | | **Microarray** | | **qPCR** | |
|  | Gene Symbol | Fold change | p value | Fold change | p value | Fold change | p value | Fold change | | p value | Fold change | p value | Fold change | p value |
|  | EPHA7 | 0.93 | 0.312 | 0.96 | 0.072 | 1.39 | 0.323 | 1.14 | | 0.066 | 1.03 | 0.687 | 1.25 | 0.072 |
|  | ETS1 | 1.01 | 0.935 | 2.89 | 0.003 | 1.06 | 0.354 | 3.50 | | 0.004 | 1.17 | 0.034 | 6.94 | 0.000 |
|  | PLXNB2 | 1.11 | 0.554 | 1.27 | 0.05 | 1.59 | 0.033 | 3.38 | | 0.024 | 2.18 | 0.010 | 1.41 | 0.031 |
|  | SEMA3D | 1.20 | 0.014 | 1.39 | 0.273 | 1.01 | 0.852 | 0.90 | | 0.335 | 0.99 | 0.793 | 2.67 | 0.017 |
|  | SEMA3G | 0.85 | 0.423 | 1.60 | 0.058 | 0.90 | 0.351 | 0.94 | | 0.079 | 1.02 | 0.914 | 2.62 | 0.042 |
|  | SEMA5B | 1.17 | 0.179 | 1.11 | 0.186 | 1.04 | 0.677 | 0.57 | | 0.136 | 1.05 | 0.531 | 3.18 | 0.003 |
|  | SEMA6D | 1.01 | 0.937 | 1.85 | 0.031 | 0.97 | 0.568 | 0.78 | | 0.078 | 0.96 | 0.478 | 2.54 | 0.023 |
|  | SEMA7A | 0.98 | 0.749 | 1.08 | 0.067 | 0.94 | 0.234 | 0.89 | | 0.054 | 0.98 | 0.658 | 1.07 | 0.097 |
|  | BAD | 1.13 | 0.219 | 0.996 | 0.790 | 1.25 | 0.041 | 1.220 | | 0.090 | 1.08 | 0.185 | 0.816 | 0.059 |
|  | BDNF | 1.01 | 0.925 | 2.89 | 0.004 | 1.07 | 0.416 | 0.931 | | 0.078 | 1.12 | 0.070 | 2.840 | 0.003 |
|  | FGFR1 | 1.07 | 0.416 | 1.529 | 0.068 | 0.73 | 0.027 | 1.357 | | 0.089 | 1.09 | 0.288 | 1.212 | 0.082 |
|  | PRKACA | 1.07 | 0.717 | 0.941 | 0.487 | 1.64 | 0.0001 | 0.843 | | 0.045 | 1.88 | 0.004 | 1.209 | 0.050 |
|  | VEGFA | 0.93 | 0.349 | 1.0134 | 0.091 | 1.08 | 0.220 | 0.920 | | 0.083 | 1.27 | 0.064 | 1.339 | 0.059 |
| **Axon guidance** | EFNA3 | 1.05 | 0.804 | 1,25 | 0.062 | 1.32 | 0.027 | 1.198 | | 0.061 | 1.61 | 0.003 | 2.352 | 0.014 |
|  | EFNB3 | 1.00 | 0.948 | 0.168 | 0.000 | 1.11 | 0.038 | 0.198 | | 0.000 | 1.67 | 0.000 | 0.149 | 0.000 |
|  | EPHA1 | 1.07 | 0.392 | 0.885 | 0.588 | 1.00 | 0.980 | 1.195 | | 0.334 | 1.22 | 0.298 | 1.871 | 0.027 |
|  | EPHB2 | 0.98 | 0.925 | 0.744 | 0.028 | 1.07 | 0.308 | 0.860 | | 0.068 | 0.88 | 0.523 | 0.436 | 0.007 |
|  | EPHB6 | 1.05 | 0.731 | 0.717 | 0.042 | 0.97 | 0.729 | 1.447 | | 0.062 | 0.94 | 0.388 | 0.941 | 0.054 |
|  | MAPK3 | 1.11 | 0.611 | 1.290 | 0.072 | 1.51 | 0.002 | 1.448 | | 0.044 | 2.02 | 0.001 | 1.393 | 0.240 |
|  | ROCK2 | 0.97 | 0.654 | 2.521 | 0.005 | 0.938 | 0.181 | 2.329 | | 0.034 | 0.951 | 0.290 | 6.463 | 0.000 |
|  | SEMA3A | 1.03 | 0.660 | 1.308 | 0.043 | 0.98 | 0.639 | 1.708 | | 0.039 | 0.99 | 0.814 | 7.081 | 0.000 |
|  | SEMA3C | 1.03 | 0.848 | 0.848 | 0.12 | 0.97 | 0.832 | 1.050 | | 0.526 | 0.93 | 0.421 | 1.573 | 0.336 |
|  | SEMA3E | 0.98 | 0.725 | 1.213 | 0.694 | 0.99 | 0.862 | 1.260 | | 0.035 | 1.04 | 0.413 | 2.103 | 0.022 |
|  | SEMA3F | 1.14 | 0.100 | 1.586 | 0.026 | 0.95 | 0.359 | 1.713 | | 0.018 | 1.08 | 0.147 | 1.139 | 0.076 |
|  | SEMA4F | 0.96 | 0.611 | 1.538 | 0.034 | 0.99 | 0.895 | 1.805 | | 0.038 | 1.24 | 0.030 | 1.276 | 0.085 |
|  | SEMA5A | 0.86 | 0.110 | 0.956 | 0.067 | 0.77 | 0.019 | 0.581 | | 0.001 | 0.40 | 0.033 | 3.413 | 0.002 |
|  | SPRED1 | 1.07 | 0.439 | 1.097 | 0.156 | 0.95 | 0.257 | 1.010 | | 0.614 | 1.00 | 0.965 | 1.490 | 0.101 |
|  | SRC | 1.07 | 0.727 | 1.208 | 0.097 | 1.33 | 0.043 | 1.454 | | 0.084 | 1.45 | 0.056 | 1.040 | 0.173 |
|  | UNC5C | 1.02 | 0.817 | 0.959 | 0.202 | 0.95 | 0.685 | 1.603 | | 0.743 | 0.71 | 0.001 | 1.671 | 0.017 |

**Supplementary Table S3.** Comparison of microarray and qPCR results in Pea3-, Erm- and Er81-transfected mHypoA-2/12 cells.

| **Neurotrophin signaling and development** |  | **Pea3** | | | | **Erm** | | | | **Er81** | | | |
| --- | --- | --- | --- | --- | --- | --- | --- | --- | --- | --- | --- | --- | --- |
|  |  | **Microarray** | | **qPCR** | | **Microarray** | | **qPCR** | | **Microarray** | | **qPCR** | |
|  | Gene Symbol | Fold change | p value | Fold change | p value | Fold change | p value | Fold change | p value | Fold change | p value | Fold change | p value |
|  | Epha7 | 0.81 | 0.001 | 0.74 | 0.062 | 0.90 | 0.00 | 0.76 | 0.261 | - | - | 1.12 | 0.413 |
|  | Plxnb2 | - | - | 0.87 | 0.891 | - | - | 1.69 | 0,340 | - | - | 1.14 | 0.090 |
|  | Sema3d | 1.39 | 0.001 | 0.74 | 0.531 | - | - | 0.37 | 0.001 | 0.59 | 0.00117 | 0.31 | 0.001 |
|  | Sema3g | - | - | 1.64 | 0.515 | - | - | 2.07 | 0.003 | - | - | 3.15 | 0.009 |
|  | Sema5b | - | - | 0.54 | 0.216 | - | - | 0.68 | 0.086 | - | - | 0.51 | 0.002 |
|  | Sema6c | 1.42 | 0.000 | 1.79 | 0.351 | - | - | 1.76 | 0.619 | - | - | 1.40 | 0.138 |
|  | Sema7a | 0.72 | 0.000 | 0.35 | 0.000 | - | - | 0.54 | 0.005 | - | - | 0.75 | 0.100 |
|  | Wnt5a | - | - | 1.13 | 0.647 | - | - | 0.88 | 0.468 | 0.55 | 0.000 | 0.57 | 0.007 |
|  | Bad | 1.21 | 0.001 | 1.20 | 0.159 | - | - | 1.22 | 0.337 | - | - | 0.81 | 0.057 |
|  | Bdnf | 0.81 | 0.002 | 0.38 | 0.000 | - | - | 1.71 | 0.019 | - | - | 1.08 | 0.923 |
|  | Egfr | 1.23 | 0.009 | 0.80 | 0.213 | - | - | 1.30 | 0.200 | - | - | 1.08 | 0.895 |
|  | Fgfr1 | 0.67 | 0.000 | 0.59 | 0.020 | - | - | 2.97 | 0.000 | - | - | 2.63 | 0.010 |
|  | Gdnf | 0.39 | 0.000 | 0.39 | 0.010 | 2.22 | 0.00 | 2.42 | 0.005 | 2.80 | 0.000 | 3.85 | 0.000 |
|  | Gsk3b | 1.36 | 0.000 | 1.12 | 0.860 | 0.77 | 0.00 | 0.93 | 0.476 | 0.69 | 0.000 | 0.93 | 0.284 |
|  | Ntf3 | 1.34 | 0.002 | 1.21 | 0.067 | 0.84 | 0.00 | 0.60 | 0.018 | 0.59 | 0.000 | 0.76 | 0.023 |
|  | Plcb3 | - | - | 0.67 | 0.089 | - | - | 1.17 | 0.100 | - | - | 0.53 | 0.004 |
|  | Prkaca | - | - | 0.51 | 0.003 | - | - | 4.36 | 0.000 | - | - | 4.23 | 0.000 |
|  | Vegfa | 0.69 | 0.000 | 0.47 | 0.000 | - | - | 1.79 | 0.068 | - | - | 1.26 | 0.591 |
| **Axon guidance** | Efna1 | 1.45 | 0.000 | 1.21 | 0.322 | - | - | 1.22 | 0.222 | - | - | 1.53 | 0.347 |
|  | Efna2 | 1.23 | 0.003 | 2.61 | 0.009 | - | - | 2.42 | 0.009 | - | - | 3.66 | 0.000 |
|  | Efna3 | 1.63 | 0.000 | 1.80 | 0.005 | - | - | 2.49 | 0.044 | 1.44 | 0.002 | 1.86 | 0.036 |
|  | Efna4 | 1.21 | 0.001 | 1.38 | 0.388 | - | - | 0.91 | 0.529 | - | - | 1.26 | 0.850 |
|  | Efnb3 | 0.55 | 0.000 | 0.37 | 0.038 | - | - | 3.93 | 0.000 | - | - | 0.85 | 0.228 |
|  | Epha1 | 1.57 | 0.000 | 1.95 | 0.016 | - | - | 1.66 | 0.012 | - | - | 1.69 | 0.024 |
|  | Epha2 | 0.72 | 0.002 | 0.74 | 0.123 | - | - | 1.64 | 0.020 | - | - | 1.60 | 0.102 |
|  | Epha4 | - | - | 1.47 | 0.051 | - | - | 1.57 | 0.001 | 0.57 | 0.002 | 0.81 | 0.415 |
|  | Ephb6 | 1.61 | 0.000 | 1.46 | 0.282 | - | - | 1.33 | 0.251 | - | - | 1.57 | 0.073 |
|  | L1cam | - | - | 0.79 | 0.860 | - | - | 1.50 | 0.048 | - | - | 0.56 | 0.985 |
|  | Irs1 | - | - | 0.69 | 0.601 | 0.82 | 0.00 | 0.94 | 0.969 | - | - | 0.84 | 0.871 |
|  | Nrg1 | 0.47 | 0.000 | 0.65 | 0.041 | - | - | 1.23 | 0.211 | - | - | 0.86 | 0.662 |
|  | Rock2 | 0.74 | 0.000 | 0.65 | 0.049 | 0.71 | 0.00 | 1.22 | 0.348 | - | - | 0.81 | 0.043 |
|  | Sema3a | 1.31 | 0.005 | 0.90 | 0.185 | - | - | 1.32 | 0.169 | - | - | 0.70 | 0.020 |
|  | Sema3c | 1.29 | 0.007 | 1.07 | 0.663 | 1.47 | 0.00 | 2.15 | 0.032 | 2.02 | 0.000 | 2.31 | 0.007 |
|  | Sema3e | 1.73 | 0.000 | 0.99 | 0.621 | 0.73 | 0.00 | 0.48 | 0.026 | 0.61 | 0.000 | 0.48 | 0.036 |
|  | Sema3f | 2.24 | 0.000 | 2.02 | 0.044 | - | - | 1.40 | 0.066 | - | - | 1,03 | 0.691 |
|  | Spred1 | 1.31 | 0.005 | 0.69 | 0.004 | 0.76 | 0.00 | 1.43 | 0.429 | - | - | 1.00 | 0.306 |
|  | Src | - | - | 0.68 | 0.149 | - | - | 1.55 | 0.009 | - | - | 1.34 | 0.247 |
|  | Unc5c | 0.79 | 0.001 | 0.81 | 0.027 | 0.77 | 0.00 | 0.58 | 0.003 | 0.68 | 0.000 | 0.73 | 0.206 |

**Supplementary Table S4.** Analysis of Pea3 transcription factor binding sites was performed on the promoter regions (-1000/+100) of target genes which were investigated by ChIP assay. Promoter sequences were scanned with Promo3.0 for Pea3 binding motif and dissimilarity scores were calculated as percentage (dissimilarity score of less than 7 % is accepted as a good candidate for binding), or cut-off p value studies in JASPAR.

| Gene Symbol | EPD ID | JASPAR (cut-off p-value) | PROMO (ds%) | Sequence | Position |
| --- | --- | --- | --- | --- | --- |
| Sema5b | FP005895 | 0.01 | 4.31% | AGGATGAC | -84 / -91 |
| Sema5a | FP007782 | 0.001 | 10.54% 0.6% | CGGATGAG  AGGATGTC | -562 / -569  -568 / -575 |
| Efna3 | FP001871 | 0.01 0.001 | 6.82% | AGGATGGG CGGAAGGC | -835 / -842 -809 / -814 |
| Efnb3 | FP022661 | 0.01 | 6.82% | GGGATGTG | -964 / -971 |
| Unc5b | FP014637 | 0.01 | 4.31% | AGGATGAT | -920 / -927 |
| Bad | FP014637 | 0.001 | 6.82% | CACATCCG | -455 / -462 |
| Bad | FP014637 | 0.01 | 0.6% | AGGATGTG | -211 / -218 |
| Sema3e | FP011219 | 0.01 0.001 | 13.05% | TGCATCCC ACTTTCCT | +13 / +20 -7 / -14 |
| Sema3a | FP011221 | 0.001 | - | ATCTTCCC | -799 / -806 |
| Epha7 | FP010106 | - | 6.82% 4.31% | TACCATCC AACATCCA | -299 / -306 -278 / -285 |

**Supplementary Table S5.** Target genes regulated by Pea3 family members Pea3, Erm and Er81 in SH-SY5Y microarray assay. Genes that are regulated by Erm and Er81 are colored blue, genes that are regulated by Er81 and Pea3 are colored in red, genes regulated by Erm and Pea3 are colored in green, genes regulated by all three family members are colored in purple, and genes only regulated by Pea3 are colored in magenta.

| **Axon guidance** | | | **Neuron differentation** | | | | **Neurotrophin Signaling** | | | |
| --- | --- | --- | --- | --- | --- | --- | --- | --- | --- | --- |
| **Er81** | **Erm** | **Pea3** | **Er81** | **Erm** | **Pea3** | **Er81** | | **Erm** | **Pea3** |  |
| AGRN | AGRN |  | AGRN | AGRN |  | AKT1 | | AKT1 |  |  |
| AP2A1 | AP2A1 |  | AKT1 | AKT1 |  | AKT2 | |  |  |  |
| ARAF | ARAF |  |  |  | AIFM1 |  | | BAD |  |  |
| ARHGAP39 |  |  | ALKBH1 | ALKBH1 |  |  | | BAX |  |  |
| ARHGEF11 | ARHGEF11 |  | ANAPC2 | ANAPC2 |  | BRAF | |  |  |  |
| CACNA1G | CACNA1G | CACNA1G | APBB1 |  |  | CALM2 | |  |  |  |
| CACNB3 |  |  | ARHGEF1 | ARHGEF1 |  | CALM3 | |  |  |  |
| CAMK2G |  |  | ATP7A | ATP7A | ATP7A | CAMK2B | |  |  |  |
| CAP1 |  |  |  |  | BCL6 | CRK | | CRK |  |  |
| CDK5 |  |  | BLOC1S1 | BLOC1S1 |  | CRKL | |  |  |  |
| CFL1 | CFL1 |  | C1QL1 |  |  |  | | FOXO3 |  |  |
| COL6A2 | COL6A2 |  | CAMK1 | CAMK1 |  | GRB2 | |  |  |  |
| CSNK2B | CSNK2B |  | CAPZB | CAPZB |  | IRAK2 | |  |  |  |
| DOK4 |  |  | CDK5 | CDK5 |  |  | | IRS1 |  |  |
| EFNB3 | EFNB3 |  | CDK5RAP2 |  |  | KIDINS220 | |  |  |  |
| GRIN2D | GRIN2D |  | CFL1 | CFL1 |  | MAGED1 | |  |  |  |
| HRAS |  |  | CHRNA3 |  |  | MAPK1 | |  |  |  |
| HSP90AB1 |  |  | CPNE1 |  |  | MAPK12 | |  |  |  |
| L1CAM | L1CAM |  | CSNK1D | CSNK1D |  | MAPK13 | |  |  |  |
| LYPLA2 | LYPLA2 |  |  | DCLK1 | DCLK1 | MAPK14 | |  |  |  |
| MAPK3 | MAPK3 |  |  |  | DLX2 |  | | MAP3K5 |  |  |
| MAPK7 |  |  | DDR1 | DDR1 |  | MAPK9 | |  |  |  |
|  | MET | MET | EFNB3 | EFNB3 |  | NFKB1 | |  |  |  |
| MYH10 | MYH10 | MYH10 | EIF4G2 |  |  | NFKBIA | |  |  |  |
| MYH9 |  | MYH9 | FLOT1 | FLOT1 |  | NFKBIB | | NFKBIB | NFKBIB |  |
| NRCAM | NRCAM |  | FRYL |  | FRYL |  | | NTRK2 |  |  |
| PDLIM7 | PDLIM7 |  | GBA2 |  |  | NTRK3 | | NTRK3 | NTRK3 |  |
| PHB |  |  | GDI1 | GDI1 |  | PDPK1 | |  |  |  |
| PIP5K1C | PIP5K1C |  | HES1 | HES1 |  | PIK3CD | |  |  |  |
| PPP2R1A |  |  | HOXD9 |  |  | PIK3R1 | |  |  |  |
| PSMC2 |  |  | HRAS |  |  | PIK3R3 | | PIK3R3 | PIK3R3 |  |
| PSMC4 | PSMC4 |  | ISL1 | ISL1 |  | RAC1 | |  |  |  |
| PSMD1 | PSMD1 |  | ITF27 |  |  | RELA | | RELA |  |  |
| PSMD3 |  |  | KDM1A | KDM1A |  |  | | RHOA |  |  |
| PSME3 | PSME3 |  | KIF26A |  |  | RPS6KA3 | | RPS6KA3 |  |  |
| PSMF1 | PSMF1 |  | L1CAM | L1CAM |  | RPS6KA5 | |  |  |  |
| RAP1GAP | RAP1GAP |  | LAMB2 |  |  | SH2B3 | |  |  |  |
| RASA4 |  |  | LGALS1 |  |  | SHC1 | | SHC1 |  |  |
| RHOC | RHOC | RHOC | LRFN4 |  |  |  | | SHC2 | SHC2 |  |
| RPS6KA2 | RPS6KA2 |  | MAPK3 | MAPK3 |  | SOS1 | |  |  |  |
| SHB | SHB |  | MAPK8IP2 | MAPK8IP2 |  | YWHAE | | YWHAE |  |  |
| SHC1 | SHC1 |  | MBD1 | MBD1 |  |  | |  |  |  |
| SHC2 | SHC2 | SHC2 | MICALL1 | MICALL1 |  |  | |  |  |  |
| SPRED2 | SPRED2 |  | MYH10 | MYH10 |  |  | |  |  |  |
| SPTAN1 | SPTAN1 |  | NAPA | NAPA |  |  | |  |  |  |
| SPTBN2 | SPTBN2 |  | NCKIPSD |  |  |  | |  |  |  |
| TLN1 |  |  | NCOA1 |  |  |  | |  |  |  |
|  |  |  | NLGN4X | NLGN4X |  |  | |  |  |  |
| TUBA1C |  |  | NRCAM | NRCAM |  |  | |  |  |  |
|  |  |  | OBSL1 |  |  |  | |  |  |  |
|  |  |  | PIN1 | PIN1 |  |  | |  |  |  |
| VEGFA | VEGFA |  | PLXNB2 | PLXNB2 |  |  | |  |  |  |
|  |  |  | PRKCSH | PRKCSH |  |  | |  |  |  |
|  |  |  | PTN |  |  |  | |  |  |  |
|  |  |  | RAP1GAP | RAP1GAP |  |  | |  |  |  |
|  |  |  | SF3A2 |  |  |  | |  |  |  |
|  |  |  | SLITRK6 | SLITRK6 |  |  | |  |  |  |
|  |  |  | SLC9A3R1 | SLC9A3R1 |  |  | |  |  |  |
|  |  |  | SPTAN1 | SPTAN1 |  |  | |  |  |  |
|  |  |  | SRGAP2 | SRGAP2 |  |  | |  |  |  |
|  |  |  | SSH2 | SSH2 |  |  | |  |  |  |
|  |  |  | SSH3 | SSH3 |  |  | |  |  |  |
|  |  |  | STAT3 | STAT3 |  |  | |  |  |  |
|  |  |  | STMN3 | STMN3 |  |  | |  |  |  |
|  |  |  | STXBP1 | STXBP1 |  |  | |  |  |  |
|  |  |  | TRAPPC9 |  |  |  | |  |  |  |
|  |  |  | UNC13A | UNC13A |  |  | |  |  |  |
|  |  |  | USP21 |  |  |  | |  |  |  |
|  |  |  | VEGFA | VEGFA |  |  | |  |  |  |
|  |  |  | ZNF335 | ZNF335 |  |  | |  |  |  |

**Supplementary Table S6.** Nervous system-related target genes regulated by Pea3 family members Pea3, Erm and Er81 in mHypoA2/12 microarray assay. Genes that are regulated by Erm and Er81 are colored blue, genes that are regulated by Er81 and Pea3 are colored in red, genes only regulated by Pea3 are colored in magenta.

| **Axon guidance** | | | **Neuron differentation** | | | **Neurotrophin Signaling** | | |  |
| --- | --- | --- | --- | --- | --- | --- | --- | --- | --- |
| **Er81** | **Erm** | **Pea3** | **Er81** | **Erm** | **Pea3** | **Er81** | **Erm** | **Pea3** | |
|  | ACTR3 |  |  |  | ABL2 |  |  | AKT2 | |
|  | ALCAM |  |  |  | ADAMTS1 |  |  | AKT3 | |
|  | ANGPT1 |  |  |  | ADCY1 |  |  | ATF4 | |
|  | ARPC5 |  |  |  | ADM |  |  | BAD | |
| BMPR2 |  |  |  |  | ADNP |  | BAX |  | |
| CALM1 | CALM1 |  |  |  | ADNP2 |  |  | BDNF | |
|  | CALM2 |  |  |  | ADRA2B |  |  | CALM1 | |
|  |  | CAMK2B |  |  | AGTPBP1 |  |  | CALM2 | |
|  | COL4A3 |  |  |  | AIFM1 |  |  | CALM3 | |
| COL4A4 | COL4A4 |  |  | ALCAM |  |  |  | CAMK2A | |
| COL4A5 | COL4A5 |  | ALKBH1 |  | ALKBH1 |  |  | CAMK2B | |
|  | COL6A1 |  |  |  | ANKRD1 |  |  | CAMK2D | |
|  | COL6A2 |  | APOA5 |  |  | CAMK4 |  |  | |
|  | COL6A3 |  | APOD |  | APOD |  |  | CRKL | |
|  | DPYSL2 | DPYSL2 |  |  | APOE |  |  | FOXO3 | |
|  | DPYSL3 |  |  | APP | APP |  |  | GRB2 | |
|  | DUSP1 |  |  |  | AREG |  |  | GSK3B | |
|  |  | EFNA1 |  |  | ARF1 |  |  | HRAS | |
|  |  | EFNA2 |  | ARF4 |  |  |  | JUN | |
|  |  | EFNA3 |  |  | ARFGEF1 | IRAK3 |  | KIDINS220 | |
|  |  | EFNA4 |  |  | ARHGAP4 |  |  | MAGED1 | |
|  |  | EPHA1 | ARTN |  |  |  |  | MAP2K5 | |
| EPHA4 |  |  | ARX | ARX | ARX | MAP3K5 |  |  | |
|  | EPHA7 |  |  |  | ASAP1 |  | MAPK12 | MAPK12 | |
|  |  | EPHB2 | ASPM | ASPM | ASPM |  |  | MAPK14 | |
|  |  | EPHB4 |  | ATAT1 | ATAT1 |  |  | MAPK9 | |
|  |  | EPHB6 |  |  | ATF1 |  |  | NFKBIA | |
|  | EVL |  |  | ATF5 |  | NFKB1 | NFKB1 |  | |
|  |  | FES |  |  | ATL1 | NFKBIA | NFKBIA |  | |
| GSK3B | GSK3B | GSK3B |  |  | AXIN1 | NFKBIB | NFKBIB |  | |
|  |  | HRAS |  | B2M | B2M | NFKBIE | NFKBIE |  | |
|  | IQGAP1 |  |  |  | B4GAT1 |  |  | NGF | |
|  | IRS1 |  |  |  | BAIAP2 |  |  | NRAS | |
| ITGB3 |  |  |  |  | BBS4 |  |  | NTF3 | |
|  | KBTBD7 |  |  |  | BCL11A | NTRK3 | NTRK3 | NTRK3 | |
| KDR |  |  | BCL6 |  | BCL6 |  |  | PIK3CA | |
|  | LAMC1 |  | BEND6 | BEND6 |  |  | PIK3R1 |  | |
|  |  | LIMK1 |  |  | BDNF |  |  | PIK3R2 | |
| MET2 |  |  |  | BHLHB9 | BHLHB9 | PIK3R5 | PIK3R5 | PIK3R5 | |
|  | MYL9 |  |  |  | BLOC1S1 |  |  | PLCG2 | |
|  | MYL12A |  |  |  | BMP4 |  |  | PRDM4 | |
|  | MYL12B |  | BMPR2 |  |  |  |  | RAC1 | |
| MYH9 | MYH9 |  | BRSK1 |  | BRSK1 |  |  | RELA | |
| MYH14 |  |  |  |  | CACNA1A |  |  | RHOA | |
|  | NCAM1 |  |  |  | CALR |  |  | RPS6KA3 | |
|  |  | NCK1 |  |  | CALU | SH2B2 | SH2B2 | SH2B2 | |
|  |  | NFATC4 |  |  | CAMK2B |  | SH2B3 | SH2B3 | |
| NGEF | NGEF |  |  |  | CAMSAP1 |  |  | SHC1 | |
|  | NRAS | NRAS | CAMSAP3 |  |  |  |  | SHC2 | |
|  | NRP1 |  |  |  | CBLN1 |  |  | TRAF6 | |
| PAK3 |  | PAK3 |  | CCDC66 | CCDC66 |  |  | YWHAE | |
| PDGFA | PDGFA |  |  |  | CCDC88A |  |  |  | |
| PDGFRB |  |  |  |  | CCK |  |  |  | |
|  |  | PDK1 | CCL5 |  | CCL5 |  |  |  | |
|  |  | PIK3CA | CDH11 |  | CDH11 |  |  |  | |
|  |  | PIK3R2 | CDH2 | CDH2 |  |  |  |  | |
|  |  | PIK3R3 |  |  | CDK5R1 |  |  |  | |
|  | PPP2R5C |  |  |  | CDK5RAP1 |  |  |  | |
|  |  | PPP3R1 |  |  | CDK5RAP2 |  |  |  | |
|  | PRKACB |  |  |  | CDK5RAP3 |  |  |  | |
|  | PRKCA |  |  |  | CDON |  |  |  | |
| PRNP | PRNP |  |  |  | CEL2R3 |  |  |  | |
|  |  | RAC1 |  |  | CEP290 |  |  |  | |
|  | RGMB |  |  |  | CHD5 |  |  |  | |
|  |  | RHOA |  | CIB1 | CIB1 |  |  |  | |
|  | ROCK2 |  | CLU | CLU | CLU |  |  |  | |
|  | RPS6KA2 |  |  |  | CMSAP3 |  |  |  | |
|  | RPS6KA3 |  | CNGB1 |  | CNGB1 |  |  |  | |
|  |  | RYK |  |  | CNP |  |  |  | |
|  | SCN3A |  |  |  | CNTF |  |  |  | |
|  | SDC2 |  | CNTNAP1 |  | CNTNAP1 |  |  |  | |
| SRGAP3 |  |  |  |  | COBL |  |  |  | |
|  |  | SEMA3A | COL25A1 |  | COL25A1 |  |  |  | |
|  |  | SEMA3C |  |  | COPS2 |  |  |  | |
| SEMA3D |  | SEMA3D |  |  | CPNE5 |  |  |  | |
| SEMA3E | SEMA3E | SEMA3E |  |  | CRABP2 |  |  |  | |
|  |  | SEMA3F |  |  | CREB1 |  |  |  | |
|  |  | SEMA6C | CSF1R |  |  |  |  |  | |
|  | SHC1 |  |  |  | CSNK1E |  |  |  | |
|  |  | SLIT2 |  |  | CTNNA2 |  |  |  | |
|  |  | SMO |  |  | CTNNB1 |  |  |  | |
|  | SPRED1 |  | CTNND2 | CTNND2 | CTNND2 |  |  |  | |
| SRGAP3 | SRGAP3 | SRGAP3 |  |  | CUL4B |  |  |  | |
| UNC5C | UNC5C |  | CUX1 | CUX1 | CUX1 |  |  |  | |
| VCL | VCL |  |  |  | CXCL12 |  |  |  | |
| WNT5A |  |  |  | CYB5D2 | CYB5D2 |  |  |  | |
|  |  |  |  | DAG1 | DAG1 |  |  |  | |
|  |  |  |  |  | DCLK1 |  |  |  | |
|  |  |  |  |  | DDIT4 |  |  |  | |
|  |  |  |  |  | DDX6 |  |  |  | |
|  |  |  |  |  | DHFR |  |  |  | |
|  |  |  | DICER1 | DICER1 |  |  |  |  | |
|  |  |  |  |  | DIXDC1 |  |  |  | |
|  |  |  |  |  | DLX1 |  |  |  | |
|  |  |  |  |  | DLX2 |  |  |  | |
|  |  |  |  | DPYSL2 | DPYSL2 |  |  |  | |
|  |  |  |  | DPYSL3 | DPYSL3 |  |  |  | |
|  |  |  | DTNBP1 |  |  |  |  |  | |
|  |  |  |  | DYLNLL2 |  |  |  |  | |
|  |  |  |  |  | EEF2K |  |  |  | |
|  |  |  |  |  | EFEMP1 |  |  |  | |
|  |  |  |  |  | EFNA1 |  |  |  | |
|  |  |  |  |  | EFNA2 |  |  |  | |
|  |  |  | EFNA3 |  | EFNA3 |  |  |  | |
|  |  |  |  |  | EFNA4 |  |  |  | |
|  |  |  |  |  | EFNB3 |  |  |  | |
|  |  |  |  |  | EGR2 |  |  |  | |
|  |  |  | EIF2AK4 |  |  |  |  |  | |
|  |  |  |  | ENC1 | ENC1 |  |  |  | |
|  |  |  |  |  | EPHA2 |  |  |  | |
|  |  |  | EPHA4 |  |  |  |  |  | |
|  |  |  |  | EPHA7 | EPHA7 |  |  |  | |
|  |  |  | EPHB2 |  | EPHB2 |  |  |  | |
|  |  |  |  |  | EPOR |  |  |  | |
|  |  |  |  |  | ERBB2 |  |  |  | |
|  |  |  | ETV5 |  |  |  |  |  | |
|  |  |  |  | EVL | EVL |  |  |  | |
|  |  |  |  |  | EZH2 |  |  |  | |
|  |  |  |  |  | FAIM2 |  |  |  | |
|  |  |  | FAS |  | FAS |  |  |  | |
|  |  |  |  | FBXW8 |  |  |  |  | |
|  |  |  |  |  | FBXO31 |  |  |  | |
|  |  |  |  |  | FES |  |  |  | |
|  |  |  |  | FEZ1 |  |  |  |  | |
|  |  |  |  |  | FEZ2 |  |  |  | |
|  |  |  |  |  | FGFR1 |  |  |  | |
|  |  |  |  |  | FKBP1B |  |  |  | |
|  |  |  |  | FKBP4 |  |  |  |  | |
|  |  |  | FLOT1 |  | FLOT1 |  |  |  | |
|  |  |  | FLRT1 |  |  |  |  |  | |
|  |  |  | FMN1 |  |  |  |  |  | |
|  |  |  |  |  | FMOD |  |  |  | |
|  |  |  | FMR1 | FMR1 |  |  |  |  | |
|  |  |  |  |  | FOXD1 |  |  |  | |
|  |  |  | FOXP1 |  | FOXP1 |  |  |  | |
|  |  |  | FRY |  | FRY |  |  |  | |
|  |  |  | FUOM |  |  |  |  |  | |
|  |  |  | FZD1 | FZD1 |  |  |  |  | |
|  |  |  | FZD2 | FZD2 | FZD2 |  |  |  | |
|  |  |  |  |  | FZD8 |  |  |  | |
|  |  |  | GABRB1 |  |  |  |  |  | |
|  |  |  |  |  | GBA2 |  |  |  | |
|  |  |  | GDNF |  | GDNF |  |  |  | |
|  |  |  |  |  | GOLGA4 |  |  |  | |
|  |  |  |  |  | GORASP1 |  |  |  | |
|  |  |  |  |  | GPR37L1 |  |  |  | |
|  |  |  | GPRC5B |  | GPRC5B |  |  |  | |
|  |  |  |  |  | GRB2 |  |  |  | |
|  |  |  |  |  | GRIP1 |  |  |  | |
|  |  |  |  |  | GRN |  |  |  | |
|  |  |  | GSK3B | GSK3B | GSK3B |  |  |  | |
|  |  |  |  |  | HDAC5 |  |  |  | |
|  |  |  |  |  | HDAC9 |  |  |  | |
|  |  |  |  |  | HIPK2 |  |  |  | |
|  |  |  | HMGB1 | HMGB1 | HMGB1 |  |  |  | |
|  |  |  |  |  | HNRNPK |  |  |  | |
|  |  |  |  |  | HRAS |  |  |  | |
|  |  |  |  |  | ID1 |  |  |  | |
|  |  |  | ID2 | ID2 | ID2 |  |  |  | |
|  |  |  |  |  | IER2 |  |  |  | |
|  |  |  | IFRD1 |  | IFRD1 |  |  |  | |
|  |  |  |  |  | IFT20 |  |  |  | |
|  |  |  | IGSF9 |  |  |  |  |  | |
|  |  |  |  |  | IL1RAPL1 |  |  |  | |
|  |  |  | IL15RA |  |  |  |  |  | |
|  |  |  | IL6 |  | IL6 |  |  |  | |
|  |  |  |  | ILK |  |  |  |  | |
|  |  |  |  |  | IMPACT |  |  |  | |
|  |  |  |  |  | INHBA |  |  |  | |
|  |  |  |  |  | INPP5E |  |  |  | |
|  |  |  |  | IQGAP1 |  |  |  |  | |
|  |  |  |  |  | IST1 |  |  |  | |
|  |  |  |  |  | ITGB1 |  |  |  | |
|  |  |  |  |  | ITPKA |  |  |  | |
|  |  |  |  |  | JAG1 |  |  |  | |
|  |  |  |  |  | JAK2 |  |  |  | |
|  |  |  |  |  | JUN |  |  |  | |
|  |  |  |  |  | KAT2B |  |  |  | |
|  |  |  | KATNA1 |  |  |  |  |  | |
|  |  |  | KCTD11 |  | KCTD11 |  |  |  | |
|  |  |  |  |  | KIDINS220 |  |  |  | |
|  |  |  |  | KIF20B | KIF20B |  |  |  | |
|  |  |  |  |  | KIF26B |  |  |  | |
|  |  |  | KIF3A |  |  |  |  |  | |
|  |  |  |  |  | KIF5C |  |  |  | |
|  |  |  |  |  | KIRREL3 |  |  |  | |
|  |  |  |  |  | KNDC1 |  |  |  | |
|  |  |  |  | LAMA2 |  |  |  |  | |
|  |  |  |  |  | LAMB1 |  |  |  | |
|  |  |  |  |  | LDB1 |  |  |  | |
|  |  |  |  |  | LGI4 |  |  |  | |
|  |  |  | LIF |  |  |  |  |  | |
|  |  |  |  |  | LIMK1 |  |  |  | |
|  |  |  |  |  | LINGO4 |  |  |  | |
|  |  |  | LINGO4 | LINGO4 |  |  |  |  | |
|  |  |  | LRFN1 |  | LRFN1 |  |  |  | |
|  |  |  |  |  | LRFN3 |  |  |  | |
|  |  |  |  |  | LRP6 |  |  |  | |
|  |  |  | LRP8 |  | LRP8 |  |  |  | |
|  |  |  | LRPG |  |  |  |  |  | |
|  |  |  |  |  | LRRC38 |  |  |  | |
|  |  |  |  |  | LRRC4C |  |  |  | |
|  |  |  |  | LSM1 |  |  |  |  | |
|  |  |  | LST1 |  | LST1 |  |  |  | |
|  |  |  |  |  | MAG |  |  |  | |
|  |  |  |  |  | MAP15 |  |  |  | |
|  |  |  | MAP2 | MAP2 |  |  |  |  | |
|  |  |  |  |  | MAP6 |  |  |  | |
|  |  |  |  |  | MAPK8IP3 |  |  |  | |
|  |  |  |  |  | MAPK9 |  |  |  | |
|  |  |  |  |  | MAPT |  |  |  | |
|  |  |  |  |  | MARCK5 |  |  |  | |
|  |  |  |  |  | MATN2 |  |  |  | |
|  |  |  |  |  | MBD1 |  |  |  | |
|  |  |  |  |  | MBP |  |  |  | |
|  |  |  | MCOLN3 |  | MCOLN3 |  |  |  | |
|  |  |  |  |  | MECP2 |  |  |  | |
|  |  |  |  |  | MED1 |  |  |  | |
|  |  |  | MEF2C | MEF2C |  |  |  |  | |
|  |  |  | MEG3 |  |  |  |  |  | |
|  |  |  |  |  | MIB1 |  |  |  | |
|  |  |  | MICALL2 |  | MICALL2 |  |  |  | |
|  |  |  |  |  | MICALLI1 |  |  |  | |
|  |  |  |  |  | MOB2 |  |  |  | |
|  |  |  |  |  | MTCH1 |  |  |  | |
|  |  |  |  | MTMR2 |  |  |  |  | |
|  |  |  |  |  | MTOR |  |  |  | |
|  |  |  |  |  | MTR |  |  |  | |
|  |  |  | MUL1 |  | MUL1 |  |  |  | |
|  |  |  | MYCN |  |  |  |  |  | |
|  |  |  |  |  | MYH10 |  |  |  | |
|  |  |  |  |  | MYO7A |  |  |  | |
|  |  |  |  |  | NAPA |  |  |  | |
|  |  |  |  |  | NBL1 |  |  |  | |
|  |  |  |  | NCAM1 |  |  |  |  | |
|  |  |  |  |  | NCK2 |  |  |  | |
|  |  |  |  |  | NCKIPSD |  |  |  | |
|  |  |  |  |  | NCOA1 |  |  |  | |
|  |  |  |  | NCS1 |  |  |  |  | |
|  |  |  |  |  | NDEL1 |  |  |  | |
|  |  |  |  |  | NDNF |  |  |  | |
|  |  |  |  |  | NEDD4L |  |  |  | |
|  |  |  | NEPRO |  | NEPRO |  |  |  | |
|  |  |  |  |  | NFASC |  |  |  | |
|  |  |  |  |  | NFATC4 |  |  |  | |
|  |  |  |  |  | NFIB |  |  |  | |
|  |  |  |  |  | NGF |  |  |  | |
|  |  |  | NGEF | NGEF |  |  |  |  | |
|  |  |  |  |  | NGRN |  |  |  | |
|  |  |  |  |  | NKX2-2 |  |  |  | |
|  |  |  |  |  | NME1 |  |  |  | |
|  |  |  |  |  | NOG |  |  |  | |
|  |  |  |  |  | NOTCH3 |  |  |  | |
|  |  |  |  |  | NPTN |  |  |  | |
|  |  |  |  |  | NR2F6 |  |  |  | |
|  |  |  |  | NRAS | NRAS |  |  |  | |
|  |  |  |  | NR2F6 |  |  |  |  | |
|  |  |  |  |  | NRCAM |  |  |  | |
|  |  |  |  |  | NRG1 |  |  |  | |
|  |  |  | NRN1 | NRN1 |  |  |  |  | |
|  |  |  |  |  | NRN1L |  |  |  | |
|  |  |  |  | NRP1 |  |  |  |  | |
|  |  |  |  |  | NRP2 |  |  |  | |
|  |  |  |  |  | NRTN |  |  |  | |
|  |  |  |  |  | NRXN3 |  |  |  | |
|  |  |  | NTF3 | NTF3 | NTF3 |  |  |  | |
|  |  |  | NTRK3 |  | NTRK3 |  |  |  | |
|  |  |  |  |  | NUMB |  |  |  | |
|  |  |  |  |  | NUMBL |  |  |  | |
|  |  |  |  |  | OGDH |  |  |  | |
|  |  |  |  |  | OGN |  |  |  | |
|  |  |  |  |  | OLFM1 |  |  |  | |
|  |  |  |  |  | OMD |  |  |  | |
|  |  |  |  |  | ONECUT2 |  |  |  | |
|  |  |  |  |  | OTX2 |  |  |  | |
|  |  |  |  |  | PACSIN1 |  |  |  | |
|  |  |  |  | PAFAH1B1 | PAFAH1B1 |  |  |  | |
|  |  |  | PAK3 |  | PAK3 |  |  |  | |
|  |  |  |  |  | PARD3 |  |  |  | |
|  |  |  |  |  | PARK2 |  |  |  | |
|  |  |  | PAX2 |  |  |  |  |  | |
|  |  |  | PBX1 | PBX1 |  |  |  |  | |
|  |  |  |  |  | PCSK9 |  |  |  | |
|  |  |  |  | PDLIM5 | PDLIM5 |  |  |  | |
|  |  |  |  |  | PHLDA1 |  |  |  | |
|  |  |  |  |  | PIGT |  |  |  | |
|  |  |  |  |  | PMP22 |  |  |  | |
|  |  |  | PPT1 | PPT1 |  |  |  |  | |
|  |  |  |  |  | PREX2 |  |  |  | |
|  |  |  |  | PRRX1 | PRRX1 |  |  |  | |
|  |  |  |  |  | PTEN |  |  |  | |
|  |  |  |  |  | PTK2B |  |  |  | |
|  |  |  | PTN |  | PTN |  |  |  | |
|  |  |  |  |  | PTPN5 |  |  |  | |
|  |  |  |  |  | PTPRF |  |  |  | |
|  |  |  |  |  | PTPRK |  |  |  | |
|  |  |  | PTPRZ1 | PTPRZ1 |  |  |  |  | |
|  |  |  |  |  | RAB10 |  |  |  | |
|  |  |  | RAB3A |  | RAB3A |  |  |  | |
|  |  |  |  |  | RAC1 |  |  |  | |
|  |  |  |  |  | RANBP9 |  |  |  | |
|  |  |  | RAP2A | RAP2A |  |  |  |  | |
|  |  |  |  |  | RAPGEF2 |  |  |  | |
|  |  |  |  |  | RAPH1 |  |  |  | |
|  |  |  |  |  | RB1 |  |  |  | |
|  |  |  |  |  | RDH13 |  |  |  | |
|  |  |  |  |  | REST |  |  |  | |
|  |  |  |  |  | RHOA |  |  |  | |
|  |  |  | RIMS1 |  | RIMS1 |  |  |  | |
|  |  |  | RND1 |  | RND1 |  |  |  | |
|  |  |  | ROBO3 |  |  |  |  |  | |
|  |  |  |  |  | ROM1 |  |  |  | |
|  |  |  |  |  | RORA |  |  |  | |
|  |  |  |  |  | RRN3 |  |  |  | |
|  |  |  |  |  | RSPO2 |  |  |  | |
|  |  |  |  |  | RTN4 |  |  |  | |
|  |  |  |  |  | RTN4R |  |  |  | |
|  |  |  |  |  | RUNX1 |  |  |  | |
|  |  |  |  |  | RUNX2 |  |  |  | |
|  |  |  |  |  | RYK |  |  |  | |
|  |  |  | SATB2 |  |  |  |  |  | |
|  |  |  |  | SDC2 | SDC2 |  |  |  | |
|  |  |  |  |  | SDC4 |  |  |  | |
|  |  |  |  |  | SECISBP2 |  |  |  | |
|  |  |  |  |  | SEMA3A |  |  |  | |
|  |  |  | SEMA3C |  | SEMA3C |  |  |  | |
|  |  |  | SEMA3D |  | SEMA3D |  |  |  | |
|  |  |  | SEMA3E | SEMA3E | SEMA3E |  |  |  | |
|  |  |  |  |  | SEMA3F |  |  |  | |
|  |  |  |  |  | SEMA4A |  |  |  | |
|  |  |  |  |  | SEMA6C |  |  |  | |
|  |  |  |  |  | SEMA7A |  |  |  | |
|  |  |  |  | SERPINF1 | SERPINF1 |  |  |  | |
|  |  |  |  |  | SGK1 |  |  |  | |
|  |  |  |  | SHC1 | SHC1 |  |  |  | |
|  |  |  |  |  | SIPA1L1 |  |  |  | |
|  |  |  | SKIL |  | SKIL |  |  |  | |
|  |  |  | SLC11A2 |  |  |  |  |  | |
|  |  |  |  |  | SLC1A3 |  |  |  | |
|  |  |  |  |  | SLC9A3R1 |  |  |  | |
|  |  |  |  |  | SLIT2 |  |  |  | |
|  |  |  |  | SMO | SMO |  |  |  | |
|  |  |  |  |  | SMURF1 |  |  |  | |
|  |  |  |  | SNAPIN | SNAPIN |  |  |  | |
|  |  |  | SOD2 |  |  |  |  |  | |
|  |  |  | SOX4 |  |  |  |  |  | |
|  |  |  |  |  | SOX9 |  |  |  | |
|  |  |  |  |  | SPG11 |  |  |  | |
|  |  |  | SPHK1 |  | SPHK1 |  |  |  | |
|  |  |  | SPINK5 | SPINK5 | SPINK5 |  |  |  | |
|  |  |  |  |  | SPP1 |  |  |  | |
|  |  |  | STAT3 |  |  |  |  |  | |
|  |  |  |  |  | STK11 |  |  |  | |
|  |  |  |  | STMN1 | STMN1 |  |  |  | |
|  |  |  | STMN4 |  | STMN4 |  |  |  | |
|  |  |  | STX3 |  | STX3 |  |  |  | |
|  |  |  |  |  | STXBP1 |  |  |  | |
|  |  |  |  |  | TBC1D24 |  |  |  | |
|  |  |  |  |  | TBX20 |  |  |  | |
|  |  |  | TBX6 |  | TBX6 |  |  |  | |
|  |  |  | TCF12 |  | TCF12 |  |  |  | |
|  |  |  | TCF4 | TCF4 | TCF4 |  |  |  | |
|  |  |  | TCTN1 |  | TCTN1 |  |  |  | |
|  |  |  |  |  | TGFB2 |  |  |  | |
|  |  |  |  |  | THOC2 |  |  |  | |
|  |  |  | THY1 | THY1 |  |  |  |  | |
|  |  |  | TLE6 |  | TLE6 |  |  |  | |
|  |  |  | TLX2 |  |  |  |  |  | |
|  |  |  | TMEM106B | TMEM106B |  |  |  |  | |
|  |  |  |  | TMEM30A |  |  |  |  | |
|  |  |  |  |  | TNC |  |  |  | |
|  |  |  |  |  | TNFRSF12A |  |  |  | |
|  |  |  |  |  | TOP2B |  |  |  | |
|  |  |  |  |  | TOPORS |  |  |  | |
|  |  |  |  |  | TRAPPC9 |  |  |  | |
|  |  |  | TSPAN2 | TSPAN2 |  |  |  |  | |
|  |  |  |  |  | TSPO |  |  |  | |
|  |  |  |  |  | TUBB3 |  |  |  | |
|  |  |  |  |  | TULP1 |  |  |  | |
|  |  |  |  |  | TULP3 |  |  |  | |
|  |  |  |  |  | UBA6 |  |  |  | |
|  |  |  |  |  | UBE2V2 |  |  |  | |
|  |  |  |  | UBE4B | UBE4B |  |  |  | |
|  |  |  |  | UHMK1 | UHMK1 |  |  |  | |
|  |  |  |  |  | ULK2 |  |  |  | |
|  |  |  | ULK4 |  |  |  |  |  | |
|  |  |  | UNC5C | UNC5C | UNC5C |  |  |  | |
|  |  |  | USH1G |  | USH1G |  |  |  | |
|  |  |  |  |  | USP21 |  |  |  | |
|  |  |  |  | USP33 | USP33 |  |  |  | |
|  |  |  | VAMP7 | VAMP7 |  |  |  |  | |
|  |  |  |  |  | VANGL2 |  |  |  | |
|  |  |  |  | VAPA | VAPA |  |  |  | |
|  |  |  | VCL | VCL |  |  |  |  | |
|  |  |  |  |  | VEGFA |  |  |  | |
|  |  |  |  |  | VEGFD |  |  |  | |
|  |  |  |  | VLDLR | VLDLR |  |  |  | |
|  |  |  |  |  |  |  |  |  | |
|  |  |  | WDPCP |  |  |  |  |  | |
|  |  |  | WDR36 |  | WDR36 |  |  |  | |
|  |  |  |  | WEE1 |  |  |  |  | |
|  |  |  |  |  | WHRN |  |  |  | |
|  |  |  |  | WNK1 | WNK1 |  |  |  | |
|  |  |  |  |  | WNT1 |  |  |  | |
|  |  |  |  |  | WNT10A |  |  |  | |
|  |  |  |  |  | WNT11 |  |  |  | |
|  |  |  |  |  | WNT16 |  |  |  | |
|  |  |  | WNT5A |  |  |  |  |  | |
|  |  |  |  |  | WNT6 |  |  |  | |
|  |  |  | WNT7A |  | WNT7A |  |  |  | |
|  |  |  |  |  | ZEB1 |  |  |  | |
|  |  |  |  |  | ZEB2 |  |  |  | |
|  |  |  |  |  | ZFYVE27 |  |  |  | |
|  |  |  | ZMYND8 |  | ZMYND8 |  |  |  | |
|  |  |  | ZSWIM6 | ZSWIM6 |  |  |  |  | |
